# Synthetic control over orientational degeneracy of spacer cations enhances solar cell efficiency in two-dimensional perovskites

**DOI:** 10.1038/s41467-019-08980-x

**Published:** 2019-03-20

**Authors:** Jun Hu, Iain W. H. Oswald, Samuel J. Stuard, Masrur Morshed Nahid, Ninghao Zhou, Olivia F. Williams, Zhenkun Guo, Liang Yan, Huamin Hu, Zheng Chen, Xun Xiao, Yun Lin, Zhibin Yang, Jinsong Huang, Andrew M. Moran, Harald Ade, James R. Neilson, Wei You

**Affiliations:** 1 Department of Chemistry, University of North Carolina at Chapel Hill, Chapel Hill, NC 27599 USA; 2 Department of Chemistry, Colorado State University, Fort Collins, Colorado, 80523 USA; 3 Department of Physics, North Carolina State University, Raleigh, NC 27695 USA; 4 Department of Applied Physical Sciences, University of North Carolina at Chapel Hill, Chapel Hill, NC 27599 USA

## Abstract

Two-dimensional perovskites have emerged as more intrinsically stable materials for solar cells. Chemical tuning of spacer organic cations has attracted great interest due to their additional functionalities. However, how the chemical nature of the organic cations affects the properties of two-dimensional perovskites and devices is rarely reported. Here we demonstrate that the selection of spacer cations (i.e., selective fluorination of phenethylammonium) affects the film properties of two-dimensional perovskites, leading to different device performance of two-dimensional perovskite solar cells (average *n* = 4). Structural analysis reveals that different packing arrangements and orientational disorder of the spacer cations result in orientational degeneracy and different formation energies, largely explaining the difference in film properties. This work provides key missing information on how spacer cations exert influence on desirable electronic properties and device performance of two-dimensional perovskites via the weak and cooperative interactions of these cations in the crystal lattice.

## Abstract (web summary)

Two dimensional halide perovskites solar cells have attracted research interest due to their higher stability compared to three dimensional counterparts. Here Hu et al. show that fine tuning of the chemical structure of the spacer cations leads to different packing arrangements and device efficiency.

## Introduction

Two-dimensional (2D) organic–inorganic hybrid perovskites (OIHPs), with a perspective of combining properties of both inorganic frameworks and versatile organics towards creating functional hybrid materials, have been studied since the 1980s^1–9^. The inorganic framework can empower the 2D OIHPs with desirable properties such as high charge carrier mobility along the sheet-like inorganic framework^4^, and the layered structure of such 2D OIHPs enables appreciable tunability in quantum confined properties (e.g., band gap, exciton binding energy) by varying the thickness of the inorganic layer^10–12^. As to the organics, a variety of conjugated molecule based ammoniums have been incorporated into 2D OIHPs, including oligothiophenes^13^, acenes^14–16^, and fullerenes^17^. Depending upon the energy levels and band gaps, these conjugated organics can contribute to light absorption and emission, and/or facilitate charge transfer between the organic and the inorganic framework^13–16,18–21^. Given the vast design space for both organic and inorganic frameworks, more “exotic” functions have also been envisioned with 2D OIHPs, including singlet fission, up conversion, among others^8,13,18,22,23^.

The lead halide-based 2D OIHPs, which have a general formula of (RNH_3_)_2_MA_*n*–1_Pb_*n*_X_3*n*+1_, have attracted much attention as alternative photovoltaic materials because of their improved stability^24–32^. However, these 2D layered perovskites, having the insulating organic cations separating these inorganic slabs, tend to adopt an orientation where these inorganic slabs would be aligned in parallel to the substrate. This would significantly hinder the charge transport in the vertical direction and result in a lower efficiency^9,28,32^. However, 2D OIHP solar cells have recently been demonstrated with significant high efficiency via a couple of methods such as hot-casting^28^ and using additives^25,31,33–35^, due to presumably achieved vertical alignment of the inorganic slabs. Perhaps due to the easy accessibility of simple aliphatic ammoniums (e.g., butylammonium, C_4_H_9_-NH_3_^+^, BA), most 2D OIHP based solar cells of high efficiency have employed them as spacer cations^25,28,31^. However, these aliphatic ammoniums are electrically insulating and not light absorbing. Ideally, one would like to replace such aliphatic ammoniums with functional organics, for example, conjugated oligomers that would absorb a complementary portion of the solar spectrum to that of the inorganic framework. This improved light absorption could potentially increase the current of 2D OIHP based solar cells and further boost their efficiency. In fact, large conjugated oligomers (e.g., tetrathiophene, acenes) have been successfully incorporated into 2D OIHPs, yet only with the *n* = 1 phase^8,13,16^. For 2D OIHPs with *n* = 2–5, which are much more relevant to photovoltaic applications, we are not aware of any successful attempts incorporating large conjugated oligomers. Nevertheless, progress has been made with 2D OIHPs based on spacer cations with single aromatic rings, such as phenethylammonium (C_6_H_5_–CH_2_CH_2_–NH_3_^+^, PEA)^24,27,33,36^, 2-thiophenemethylammonium^34^, 3-bromobenzylammonium^35^, among others. Yet, most of these studies relied on special additives such as NH_4_SCN, MACl, etc. to achieve a high efficiency. Thus, chemical tuning of these spacer cations and its impact on the structure of 2D OIHPs as well as the device performance of related solar cells become an interesting yet under-explored direction.

Here we show that selectively monofluorinating PEA at different positions of the aromatic moiety (benzene in this case) can significantly affect the photovoltaic device efficiency of these 2D OIHPs (*n* = 4). Monofluorination of PEA is perhaps the smallest perturbation to the PEA-based spacer cations, which should not significantly change the molecule size or add additional optoelectronic functionalities. While we observe over 10% photovoltaic efficiency when 3-fluorophenethylammonium (mF1PEA) or 4-fluorophenethylammonium (pF1PEA) is used as the organic cation in 2D OIHP based solar cells, the efficiency of solar cells based on 2-fluorophenethylammonium (oF1PEA) is less than 1%. We find the observed difference in efficiency can be explained by considering three key properties of the 2D OIHP films: phase distribution, surface morphology and crystal orientation. To further understand how the organic cation would affect the structure of these 2D OIHPs, we analyze single crystals of 2D OIHPs (*n* = 1) with these fluorinated PEA cations and disclose that all crystals have a similar inorganic framework structure, yet very different organic cation packing arrangements. Specifically, the crystals with high orientational disorder of the organic cations yield poor device performance. Combined with density functional theory (DFT) calculations, we find the differences in formation energies of these compounds correlate well with variations in packing and disorder of the spacer cations. It appears that having a more favorable formation energy and less crystallographic disorder are beneficial for the device performance of these 2D OIHP based solar cells.

## Results

### Photovoltaic device performance

**Fig. 1:**
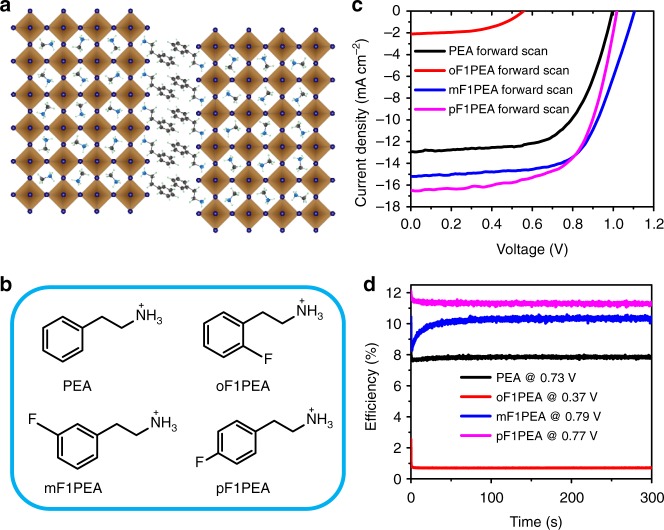
2D OIHPs with PEA fluorinated at different positions and their device characterization: **a** crystal sketch of PEA based 2D OIHP (*n* = 4); **b** molecular structures of PEA and its fluorinated derivatives in this study; **c** current-density-voltage (*J*-*V*) curves (forward scan) under 1 sun condition (AM.1.5 G) with a solar simulator; **d** device performance tracking under constant illumination and at maximum power point

We chose lead iodide based 2D OIHPs with a nominal *n* = 4 average composition (Fig. 1a) as the active layer for our solar cells with a *p*-i-*n* planar structure^28,37^. Four structurally-related large organic cations were employed to construct the 2D OIHPs: PEA, oF1PEA, mF1PEA, and pF1PEA (Fig. 1b, Supplementary Figure 1). Although these cations do not directly contribute to charge transport or light absorption by themselves^38–40^, they significantly influence the photovoltaic device performance. For example, we achieved an optimized efficiency of 7.67% with small hysteresis for devices with PEA (Fig. 1c, Supplementary Figure 2, 3 and Supplementary Table 1–3). This efficiency is comparable to or higher than literature values (for the cells fabricated without special processing conditions such as hot-casting or using additives)^24,27^ and consistent with our External Quantum Efficiency (EQE) measurement (Supplementary Figure 4 and Supplementary Note 1). When mF1PEA or pF1PEA were used instead of PEA, all key device characteristics—open circuit voltage (*V*_oc_), short circuit current (*J*_sc_) and fill factor (FF)—were noticeably improved, resulting in higher efficiency values (over 10%) (Supplementary Figure 3 and Supplementary Table 1). However, if oF1PEA was employed, the photovoltaic device showed very poor efficiency values (less than 1%). We observed certain hysteresis in our 2D OIHP based solar cells (Supplementary Figure 2 and Supplementary Table 1), which could be ascribed to trap states or ion migration^41–45^. Nevertheless, all devices maintained consistent efficiency under constant illumination and at maximum power point (Fig. 1d).

### Characterizations of 2D OIHP Films

**Fig. 2:**
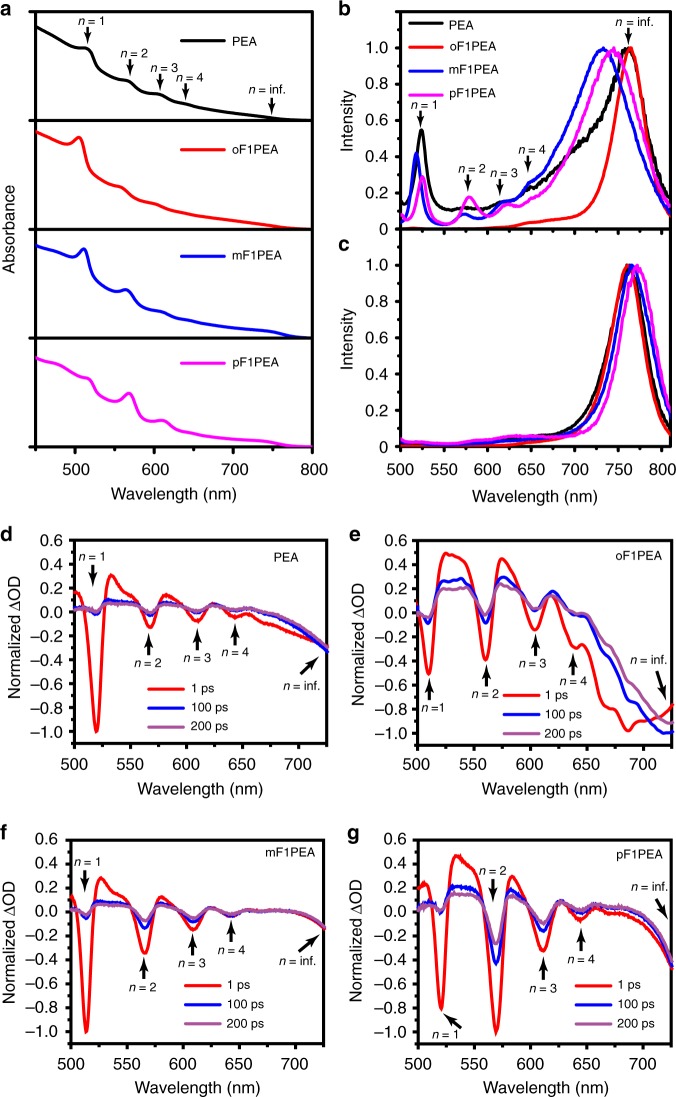
Optical properties of 2D OIHP films: **a** UV–Vis absorption; **b** normalized photoluminescence from back (glass) side, and **c** from front (air) side. Transient absorption spectrum excited from back (glass) side of 2D OIHP films based on PEA (**d**), oF1PEA (**e**), mF1PEA (**f**), and pF1PEA (**g**). The excitation wavelength was chosen at the absorption peak of the particular *n* = 1 phase for each 2D OIHP film, i.e., 517, 506, 510, and 518 nm, respectively. Numbers and arrows indicate signals from different phases

We first characterized the absorption of each film to investigate the differences in light absorption and film composition, which could account for the observed difference in device performance. For each film, we observed several excitonic peaks (Fig. 2a), corresponding to a distribution of perovskite phases with different *n*. This observation indicates that there are multiple perovskite phases in these 2D OIHP films having a nominal *n* = 4^46–49^. However, there are subtle differences when comparing these absorption spectra. For example, the absorbance of *n* = 1 phase in pF1PEA based 2D OIHP is weaker than the corresponding *n* = 1 phase in other 2D OIHPs in this series, while the 3D perovskite absorption in mF1PEA or pF1PEA based 2D OIHP was stronger than those in PEA and oF1PEA 2D OIHPs. These results indicate that the relative distribution of 2D phases having different *n* values in each film is different. Nevertheless, the overall light absorption of each film is similar in terms of the intensity and peak position, thus the light absorption cannot be the key reason to explain the large performance difference in photovoltaic devices.

We further conducted photoluminescence (PL) of each film from both back (glass) side and front (air) side as shown in Fig. 2b, c. For PEA, mF1PEA or pF1PEA based 2D OIHP, PL signals from 2D phases with different *n* values are clearly visible when excited from the substrate side (i.e., back side) (Fig. 2b). However, when excited from the air side (i.e., front side), signals from these 2D phases (*n* = 1, 2, 3, etc.) are much weaker, and the spectrum is essentially dominated by the PL from the 3D perovskite phase (Fig. 2c). We note that, when excited from the air side, the emission peaks for the 3D perovskite phases in our 2D OIHPs are slightly blue shifted when compared to the emission of normal 3D perovskites at 770 nm (Supplementary Figure 5). It may be caused by the slight twisting of the 3D crystal lattice (the twisted Pb-I-Pb angle) due to the presence of 2D phases which may impose a large internal strain in the formation of mixed perovskite films^30,50^. A blue shift of 3D phase was also observed previously when the ratio of PbI_2_ and MAI was changed, which may also occurs here^51^. However, when excited from the glass side, we observed much different emission peaks from 733 to 765 nm in the case of PEA, mF1PEA and pF1PEA 2D OIHP films (Fig. 2b). These emission peaks for “3D” perovskites are asymmetric and have significant blue-shifts due to the PL from 3D phase blended with the significant emission from large *n* phases. These observations indicate in PEA, mF1PEA, or pF1PEA based 2D OIHP films, a vertical phase distribution of multiple 2D OIHP phases occurs, where phases having smaller *n* values dominate on the glass side of the substrate while phases with larger *n* or 3D appear more on the air side. As suggested by others, this sequential phase distribution could benefit energy transfer (and/or charge transfer) across the film and lead to high device performance (Supplementary Figure 6a)^37,46–49,52^. However, for oF1PEA 2D OIHP, we only observed a strong and dominating emission peak from the 3D perovskite phase for both back side and the front side excitation (Fig. 2b, c), and these two emission spectra for oF1PEA 2D OIHP are almost identical. This suggests that the front side (i.e., air side) and back side (i.e., glass side) of the oF1PEA OIHP film are similar in terms of composition—both contain a significant amount of 3D perovskite phases, with 2D phases randomly mixed within. This scenario, i.e., a random phase distribution, would allow the 2D phases in the oF1PEA OIHP film to efficiently transfer their energy to large *n* phases and eventually to 3D phases. However, this random phase distribution in the oF1PEA 2D OIHP film would also result in charge trapping at the small band gap phases (schematically shown in Supplementary Figure 6b) and lower the *J*_sc_ and FF of the corresponding photovoltaic device. We believe this is one of the reasons to account for the extremely poor photovoltaic device performance of 2D OIHP based on oF1PEA.

To further verify the proposed phase distribution, we conducted transient absorption (TA) measurements on all four 2D OIHP films. Samples were pumped from the glass side at the wavelength of the absorption of the particular *n* = 1 phase for each 2D OIHP film. Upon excitation, at very early stage (1 ps), we observed strong bleach signal of small *n* phases (*n* = 1 or 2) and relatively weak bleach signal from 3D or large *n* components in the case of PEA, mF1PEA, and pF1PEA based 2D OIHP films (Fig. 2d–g). In contrast, oF1PEA 2D OIHP film shows strong bleach signal for 3D phase signal and relatively weak bleach signal for 2D phases (Fig. 2e). These results support our claim that for PEA, mF1PEA, and pF1PEA based 2D OIHP films, 2D phases with small *n* dominate the composition on the glass side whereas for oF1PEA, there is a significant amount of 3D phases on the glass side. The dynamics for each 2D OIHP sample also support the corresponding energy transfer mechanisms (Supplementary Figure 7 and Supplementary Note 2). The proposed phase distribution and energy transfer were further supported by characterizations of time of flight secondary ion mass spectrometry (ToF-SIMS, Supplementary Figure 8 and Supplementary Note 3) and ultraviolet photoelectron spectroscopy (UPS, Supplementary Figure 9, Supplementary Table 4 and Supplementary Note 4).

**Fig. 3:**
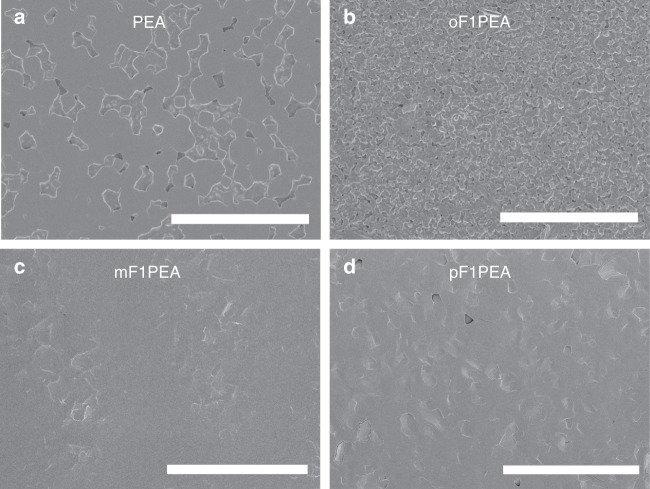
Surface morphology of 2D OIHP films: SEM images of 2D OIHP films based on **a** PEA, **b** oF1PEA, **c** mF1PEA, and **d** pF1PEA. Scale bar is 10 µm

We next investigated the film surface morphology by scanning electron microscope (SEM) (Fig. 3). While PEA 2D OIHP film is quite uniform with some visible pinholes, the oF1PEA 2D OIHP film is very rough and discontinuous. This undesirable surface morphology of the oF1PEA 2D OIHP film could lead to partially shorting of the device or bad contact between the electrodes (including interfacial layers) and the perovskite film, leading to inferior device performance (e.g., low *V*_oc_, FF). In contrast, both mF1PEA and pF1PEA 2D OIHP films are uniform without pinholes, a surface morphology conducive to the high device performance we achieved. Probing the surface roughness with atomic force microscopy (AFM) also shows a similar trend (Supplementary Figure 10), with mF1PEA and pF1PEA films show the smallest roughness (less than 20 nm). Overall, we observed the consistent relationship between the surface morphology and device performance.

**Fig. 4:**
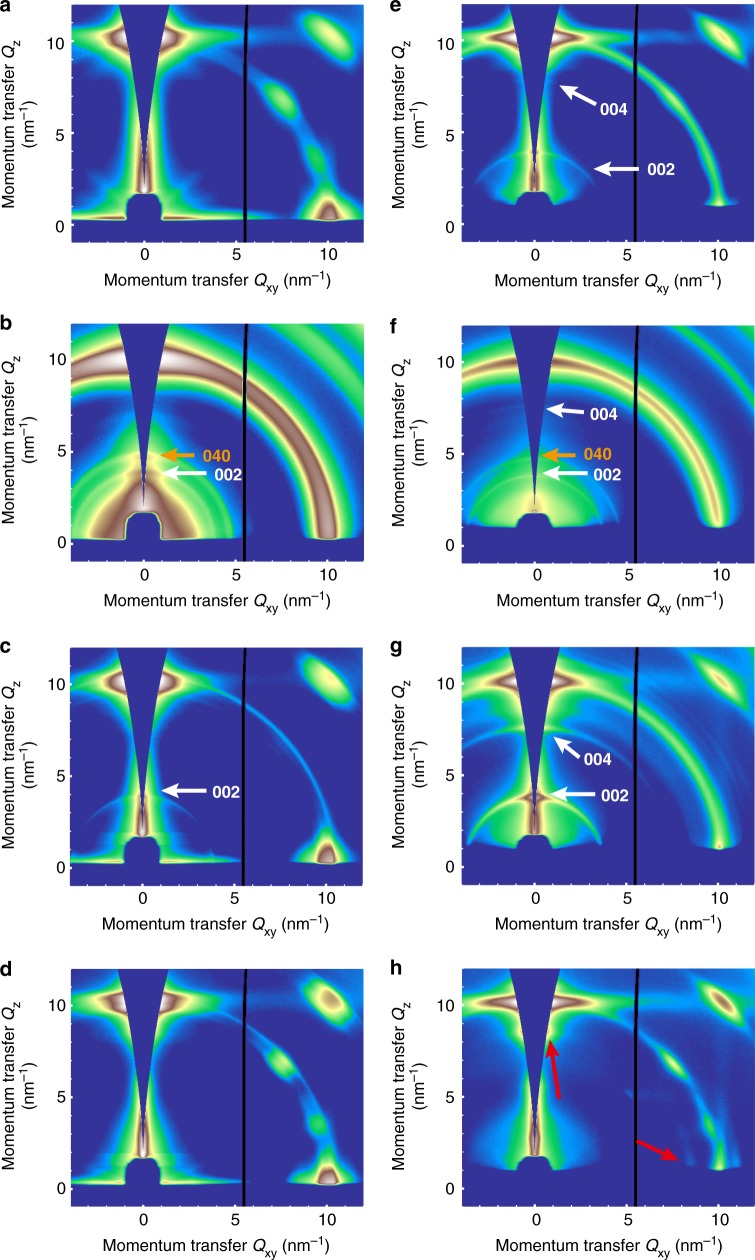
GIWAXS patterns of 2D OIHP films with PEA and fluorinated PEAs under different incident angles: GIWAXS patterns of **a**, **e** PEA, **b**, **f** oF1PEA, **c**, **g** mF1PEA, and **d**, **h** pF1PEA 2D OIHP films probing at two different X-ray incident angles; left column (**a**–**d**) shows patterns acquired at incident angle = 0.18°, probing a few nanometers of the perovskite films from the front (air) interface respectively, while right column (**e**–**h**) shows patterns acquired at incident angle = 1°, probing more into the bulk of the films. Arrows and their indices indicate distinct peaks from 2D phases: white and orange arrows show *n* = 1, and *n* = 3, and red arrows indicate peaks which are possibly due to the larger *n* values such as *n* = 4. Peaks were indexed by comparing the GIWAXS experimental patterns to a set of simulated diffraction patterns using CrystalDiffract^®^ (Version 6.7.3)

In addition to phase distribution and surface morphology, crystal orientation was also reported to be an important factor that can remarkably affect the device performance due to the anisotropic nature of 2D OIHPs^28,53,54^. To investigate the crystal orientation, we performed grazing incidence wide angle X-ray scattering (GIWAXS) on each sample. GIWAXS can elucidate all directions of orientation and we conducted incident angle-dependent experiment to probe different depth from the surface of the film^55^ (Fig. 4 and Supplementary Figure 11). A lower X-ray incident angle GIWAXS experiment, for example, 0.18°, allows the acquisition of the diffraction data from the top few nanometers of the 2D OIHP films (Fig. 4, left column), whereas a higher incident angle, for example, 1°, probes deeper into the bulk of a film (Fig. 4, right column)^56–58^. First, as a whole, our GIWAXS data for PEA, mF1PEA or pF1PEA based 2D OIHP films clearly show strong, sharp and discrete Bragg spots, indicating a highly orientated film in each case (see Supplementary Figure 11 for full GIWAXS patterns)^59–63^. On the other hand, the oF1PEA 2D OIHP film exhibits diffraction rings, suggesting no preferred orientation of crystallites^28^. This observation is also consistent with the film X-ray diffraction pattern (Supplementary Figure 12, 13 and Supplementary Discussion 1). Therefore, the smaller crystallites in the oF1PEA 2D OIHP film must be randomly distributed without a particularly preferred orientation. This random crystal orientation would hinder the charge transport across the film, further explaining the low device performance (e.g., low *J*_sc_ and FF). Second, we investigate the presence of unique 2D OIHP signatures in the GIWAXS patterns in all 2D OIHP films in this study. Venkatesan et al. have shown that 2D OIHP films exhibit diffraction peaks less than 10 nm^−1^ because the spacings associated with the low-*Q* peaks are only possible when the larger cations are present in the system^64^. Similar to their findings, at the incident angle = 0.18°, the oF1PEA (Fig. 4b) and mF1PEA (Fig. 4c) 2D OIHP films show clear signals for the *n* = 1 2D phase around *Q*_z_ = 4 nm^−1^, as indicated by the white arrows and indices. The oF1PEA 2D OIHP film has an additional peak around *Q*_z_ = 4.8 nm^−1^, which can be assigned to *n* = 3 (Fig. 4b, orange arrow and index). In sharp contrast, when probed at the same 0.18° incident angle, we do not observe any uniquely 2D OIHP peaks (no clear spots or arcs located at *Q* < 10 nm^−1^) in the PEA (Fig. 4a) and pF1PEA (Fig. 4d) 2D OIHP films. This observation of unique low-*Q* features in GIWAXS directly corroborates with the XRD findings where oF1PEA and mF1PEA 2D OIHP films show low 2*θ* peaks (Supplementary Figure 13 and Supplementary Discussion 1).

Finally, to understand the vertical distribution of these 2D phases in the 2D OIHP films, we probed all these films with higher incident angle (1°, Fig. 4e–h). Interestingly, under this condition, the PEA (Fig. 4e) and pF1PEA (Fig. 4h) 2D OIHP films do show 2D OIHP peaks at *Q* < 10 nm^−1^. In fact, such 2D OIHP peaks in the case of the mF1PEA 2D OIHP film (Fig. 4g) are now even stronger at this higher incident angle. While a few peaks for the pF1PEA 2D OIHP film (Fig. 4h, red arrows) could not be assigned to known 2D phases (*n* < 4), these peaks are still at a *Q* < 10 nm^−1^, suggesting that there might be larger *n* 2D phases (e.g., *n* = 4). The stronger intensity of 2D OIHP patterns in PEA, mF1PEA, and pF1PEA 2D OIHP films under higher incident angle indicate that there exists a mixture of 2D phases of different *n* values, and their concentrations are higher towards the back of the films. On the other hand, the oF1PEA 2D OIHP film (Fig. 4f) exhibits no obvious changes when probing at the higher angle, indicating that oF1PEA 2D OIHP film has a more random phase distribution. These results from GIWAXS for 2D OIHP with different cations strongly agrees with the different phase distribution probed by the prominent PL spectra as discussed earlier (Fig. 2b, c).

The presence of more 2D phases on the surface of mF1PEA 2D OIHP than other studied 2D OHIPs in this work, as indicated by the GIWAXS data (Fig. 4), also helps explain the relatively higher stability of mF1PEA 2D OIHP solar cell than pF1PEA, PEA, and the widely studied BA based 2D OIHP solar cells. As shown in Supplementary Figure 14, when the un-encapsulated devices were stocked under ambient condition (relative humidity around 45%), mF1PEA base 2D OIHP solar cells showed the best stability, while others showed lower stability.

### Orientational disorder from single crystal analysis

To further understand how different organic cations impact the structure and texture of these 2D OIHP films, we grew single crystals of 2D OIHPs (*n* = 1) with each of these four different cations (Supplementary Figure 15), following a procedure previously reported (details in Methods section)^65^. Though each 2D OIHP film analyzed in this study contains multiple phases (e.g., *n* = 1, 2, 3, 4, etc.), we chose to synthesize and study single crystals of the *n* = 1 phase for two reasons. First, the *n* = 1 phase is the fundamental building unit towards larger *n* number 2D phases (*n* = 2, 3, 4, etc.). Only containing the [PbI_6_] octahedra and the large cation (i.e., no methylammonium), the crystal structure of the *n* = 1 phase allows us to focus on the interaction between these inorganic octahedra and organic cations. Second, the *n* = 1 phase can be reliably grown as a pure single crystal with the least amount of dynamic disorder of organic cations or with lamellar disorder; therefore, the analyzed structure can serve as the structural input for density functional calculations.

**Fig. 5:**
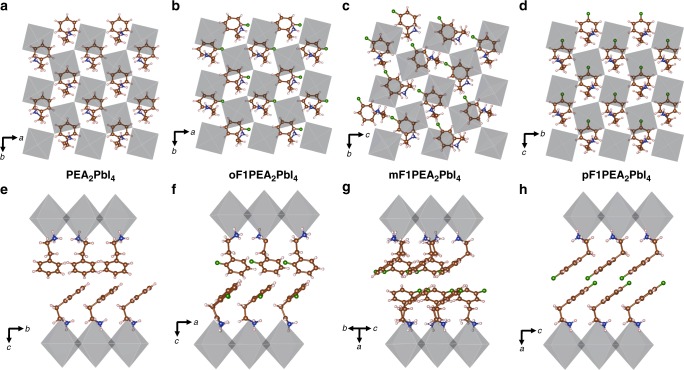
Single crystal structures of 2D OIHPs (*n* = 1) with different cations: Idealized crystal structures showing the different ordering of organic cations within a single layer for PEA (**a**) (this structure was redrawn based on the work reported by Du et al.)^38^, oF1PEA (**b**), mF1PEA (**c**), and pF1PEA (**d**). Idealized crystal structures showing the different packing arrangements within the organic interlayer gallery for PEA (**e**), oF1PEA (**f**), mF1PEA (**g**), and pF1PEA (**h**)

Single crystal structural models reveal that all three monofluorinated PEA (i.e., oF1PEA, mF1PEA, and pF1PEA) based 2D crystals are composed of sheets of corner-sharing [PbI_6_] octahedra that are separated by the organic cations (Fig. 5b-d, f-h, Supplementary Table 5, Supplementary Table 6 and Supplementary Discussion 2), similar to that seen in the PEA_2_PbI_4_ 2D crystal previously reported (Fig. 5a, e)^38^. Two of the 2D OIHP crystals, pF1PEA_2_PbI_4_ and mF1PEA_2_PbI_4_, are isostructural to their Sn analogs (4-FPEA)_2_SnI_4_ and (3-FPEA)_2_SnI_4_^66^, while oF1PEA_2_PbI_4_ adopts a lower symmetry structure (space group *P*$\bar 1$) owing to a different packing motif of the organic molecules in the interlayer gallery. The location of the fluorine atom on the PEA (ortho, meta, para) leads to changes in the packing of the bulky organic cations relative to one another with varying degrees of structural disorder. For instance, the packing of pF1PEA_2_PbI_4_ has the cations within the interlayer gallery facing the same direction in a co-aligned fashion (Fig. 5d, h), which is consistent with the Sn-analogue reported earlier^66,67^. The structure is fully ordered with no split atomic sites, indicating registry between neighboring layers is retained throughout the structure^67^. On the other hand, mF1PEA_2_PbI_4_ has a different packing motif: within a single layer of the organic interlayer gallery, neighboring aromatic moieties are rotated relative to one another to generate a herringbone configuration (Fig. 5c). Another significant difference between mF1PEA_2_PbI_4_ and pF1PEA_2_PbI_4_ is the presence of split equatorial iodine atomic positions and consequentially a splitting of the ammonium groups on the mF1PEA cations; attribution of this disorder and a logical construction of the idealized configuration is described in the Supplementary Information (Supplementary Figure 16). This disorder is similar to that observed in (3-FPEA)_2_SnI_4_^66^ where the splitting of sites was attributed to loss of registry between inorganic sheets within the material. Lastly, oF1PEA_2_PbI_4_ has yet another packing motif that is similar to PEA_2_PbI_4_. Within the organic interlayer gallery, the organic cations in the top layer are co-aligned facing the same direction (Fig. 5b), but the neighboring bottom-most layer cations are then rotated ~90° relative to the top-most layer (Fig. 5f). Additionally, the crystal structure of oF1PEA_2_PbI_4_ reveals a lack of ordering of the organic bilayer perpendicular to the planes (Supplementary Figure 17, 18). This is different from PEA_2_PbI_4_, which does have ordering of the organic cations. This indicates that oF1PEA_2_PbI_4_ has the most orientational disorder of the three monofluorinated PEA based 2D crystals. Therefore, we propose that during the film formation of the oF1PEA based 2D OIHP film, the lack of ordering of the organic bilayer would cause the formation of small crystal domains and may also play a part in why the film morphology and subsequent device performance was so drastically lower than either PEA, pF1PEA, or mF1PEA 2D OIHP films. We will further quantify this difference through the formation energy difference conducted by DFT calculation (*vide infra*).

### DFT calculations

**Fig. 6:**
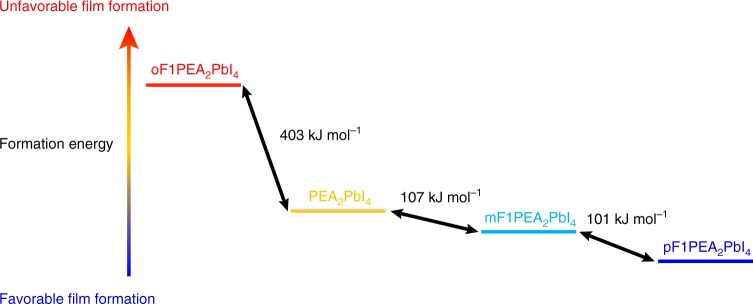
Relative formation energy difference between different 2D OIHPs (*n* = 1). DFT calculation of formation energy for PEA_2_PbI_4_, oF1PEA_2_PbI_4_, mF1PEA_2_PbI_4_, and pF1PEA_2_PbI_4_

The analyzed structures of *n* = 1 2D crystals allowed us to conduct DFT calculations of the formation energies of these *n* = 1 2D OIHPs (i.e., the total energy difference between the final perovskite structure and the starting aryl-ammonium iodide and lead iodide)^27^. Details of DFT calculation parameters are shown in Supplementary Note 5. The relative formation energies are shown in Fig. 6 and Supplementary Table 7. For mF1PEA_2_PbI_4_ and pF1PEA_2_PbI_4_, their formation energies are close, and more favorable towards the formation of the *n* = 1 2D crystal than that of PEA_2_PbI_4_; in contrast, the formation energy of oF1PEA_2_PbI_4_ is the least favorable. Indeed, the trend of the calculated formation energy is consistent with the quality of crystals (e.g., the crystal size and visible defects) we obtained (pF1PEA_2_PbI_4_ ≈ mF1PEA_2_PbI_4_ > PEA_2_PbI_4_ » oF1PEA_2_PbI_4_, Supplementary Figure 15). This trend also follows the trend observed in the structural transition enthalpy determined by differential scanning calorimetry (DSC) reported by Li et al.^65^, indicating that this simulated formation energy trend is in agreement with experiments. In addition, the relatively unfavorable formation energy of oF1PEA_2_PbI_4_ can also lead to the most disordered crystal structure among these four crystals, which—again—agrees with the single crystal structure discussed earlier. Finally, this formation energy trend also matches the device performance we observed. Therefore, we propose that this different formation energy is likely what leads to the different film properties in our study in three aspects. First, with a more favorable formation energy (e.g., pF1PEA_2_PbI_4_ in Fig. 6), it is relatively easy to form large domains and results in a more compact film. For the unfavorable formation energy of oF1PEA_2_PbI_4_, there is an orientational degeneracy of the dipole formed by the fluorine substitution, leading to crystallographic disorder; as such, macroscopic defects are more likely to form and lower the device performance. Second, it has been reported that the driving force to form 2D OIHP with smaller *n* value is larger^27^. We propose that during the film formation following our fabrication procedure, the back side (i.e., glass side) of the film was heated first and quickly accumulated with the formation of the phase with small *n* values. Because of the formation of small *n* phases, the stoichiometric ratio of the precursor would shift to a phase with an even larger *n* value. As a result, the ordered phase distribution with small *n* value phase at the bottom (glass side) and larger *n* value phase on the top (air side) were formed. In the case of oF1PEA_2_PbI_4_, the formation energy is less favorable than those of the rest, which could hinder the quick formation small *n* phase at the early stage. As a result, the unfavorable disordered phase distribution would occur in the oF1PEA 2D OIHP film. Third, the small crystal domain and random orientation in oF1PEA 2D OIHP films, caused by the unfavorable formation energy and highly disordered crystal structure, would further hinder the device performance. In addition, it is worth noticing that the inorganic frameworks (i.e., the Pb-I layer) in these crystals are almost identical (e.g., bond length, Pb-I-Pb angle, etc., Supplementary Table 8). Therefore, the main difference among these crystals must be from the packing of the organic layers (i.e., packing between PEA or F1PEA). For 2D OIHP phases with a higher *n* value where the only difference is increasing the thickness of the inorganic slab, the organic spacer layers would retain the same orientational preferences due to the shapes of the fluorinated molecules, even with slight differences in octahedral tilting; therefore, we propose that the trends in formation energy of *n* = 1 phases are representative of all compounds with *n* > 1. Furthermore, given the only difference among these cations is the substitution position of the fluorine (or no fluorine in the case of PEA), we believe that the noncovalent interaction, for example, dipole interaction between these large cations, will highly influence the film properties and therefore, result in different device performance.

## Discussion

In summary, we discovered that the photovoltaic device performance of 2D OIHPs can be significantly impacted by the spacer cation chemistry and packing, as established by a seemly negligible change in the position of monofluorination on the PEA cation. In the studied series of 2D OIHP films (PEA, oF1PEA, mF1PEA, and pF1PEA), the pF1PEA based 2D OIHP showed the highest efficiency of over 10%, followed by mF1PEA (over 10%), PEA (over 7%), and oF1PEA (less than 1%). Through an orchestra of experiments, we have identified possible causes to account for the observed difference in device performances. First, all these films contain multiple 2D phases (*n* = 1, 2, 3, 4, etc.) and 3D phases, yet the distribution of these phases are different for each 2D OIHP film in this study. While three OIHP films (PEA, mF1PEA and pF1PEA) show more or less a vertical phase distribution where the small *n* phases accumulated near the substrate and 3D phases near the surface of the film, the oF1PEA OIHP film only shows a rather random distribution of 2D phases. Second, the surface of oF1PEA OIHP film is very rough and discontinuous, which is undesirable for solar cells. Third, the crystallites in the oF1PEA OIHP film are small and randomly distributed without particularly preferred orientation.

Taking one step further, the single crystal structures of related *n* = 1 2D OIHPs and DFT calculation establish the rules for packing of these substituted organic cations; such packing results in various degrees of orientational order and disorder, depending upon the nature of the organic cations, as predicted by the formation energy. These differences could be caused by the organic cation interactions (e.g., dipole–dipole interactions), which is likely the fundamental reason for the observed photovoltaic device differences among these different PEA cations based 2D OIHP films.

Furthermore, our study offers an example where chemical modification of organic cations, device performance, film morphology and crystallography are integrated to derive the structure-properties relationships. Such relationships—where weak yet cooperative interactions can still yield macroscopic differences in performance—provide a guide for the design of materials with desirable properties for functional devices. We note cations with single benzene ring like PEA lack the coveted optoelectronic functionalities; however, this work still provides valuable insight to the future design of more complex conjugated cations to achieve desirable properties for a variety of applications including photovoltaics^24,34,35^, light emitting^13,48,49^, transistors^4,66^, spin electronics^68^, etc. We believe future design of organic cations and understanding the intermolecular interactions associated with these organics, will play an essential role in further development of these 2D OIHP materials.

## Methods

### Synthesis of MAI, PEAI, oF1PEAI, mF1PEAI, and pF1PEAI

Eleven milliliter unstabilized hydroiodic acid (HI) (57 wt% in water, Sigma-Aldrich) was purified using a 0.36 M tributyl phosphate solution in chloroform, following a previous reported procedure^24^. Then the as-prepared HI was dropped into a cold 10 mL methylamine solution (40 wt% in water, Sigma-Aldrich) under stirring. The crude product was obtained by slowly evaporating the solvent under reduced pressure. Then the white precipitate was dissolved and recrystallized in ethanol and washed with ethyl ether. The product was dried under vacuum overnight.

Five milliliter phenethylammonium (Alfa Aesar, 99%) or 2-fluorophenethylamine (Acros Organics, 97%) or 3-fluorophenethylamine (Alfa Aesar, 97%) or 4-fluorophenethylamine (Sigma-Aldrich, 99%) was diluted with 5 mL ethanol. As-purified HI was dropped into an ice-cold amine solution under stirring. The crude product was precipitated during the process. Ethyl ether was then added into the solution to further precipitate the ammonium product. Then the white precipitate was re-dissolved and recrystallized with ethanol, and lastly washed with ethyl ether. The products were dried under vacuum overnight.

### Deposition of perovskite films

For 2D OIHP films, the precursor solution was made by dissolving PEAI (or oF1PEAI, mF1PEAI, pF1PEAI), MAI and PbI_2_ (99.9985%, from Alfa Aesar) in dimethylformamide (DMF) (from Sigma-Aldrich) with the molar ratio of PEAI:MAI:PbI_2_ = 2:3:4 and the mixture was stirred for at least 60 min. The concentration of Pb^2+^ was 1 M for the precursor solution used to prepare the OIHP films for all characterizations except for TA measurement, where the precursor solution adopted a concentration of 0.5 M for Pb^2+^. The 2D OIHP film was spin-coated at 5000 rpm for 20 s from the precursor solution on a substrate (glass substrates for XRD and optical characterizations; poly(3,4-ethylenedioxythiophene) polystyrene sulfonate (PEDOT:PSS) coated indium doped tin oxide (ITO) substrates for solar cells, SEM and AFM; Si wafer for ToF-SIMS; PEDOT:PSS coated Si wafer for GIWAXS) and then the resulting film was quickly transferred to a hot plate at desired temperature (65 °C for pF1PEA and 40 °C for the rest) to anneal 30 s. The spin-coating process was done in an ambient condition with a relative humidity around 10–45%.

For 3D OIHP (MAPbI_3_), film was deposited by one-step anti-solvent extraction approach. The perovskite precursor solution was prepared by dissolving 461 mg PbI_2_ and 159 mg MAI in 700 mL DMF and 78 µL dimethyl sulfoxide (DMSO). Then the MAPbI_3_ precursor solution was spun onto the substrate (glass for XRD and optical characterizations) at 2000 rpm for 2 s and 4000 rpm for 20 s, the sample was drop-casted with 0.3 mL toluene at 8 s of the second-step spin-coating. Subsequently, the sample was annealed at 65 °C for 10 min and 100 °C for 10 min

### Device fabrication and characterization

Glass substrates coated with patterned ITO (Thin Film Devices, Inc with a resistivity of 20 Ω □^−1^) were ultrasonicated in deionized water, acetone, and 2-proponal for 15 min each sequentially. The substrates were dried under a stream of nitrogen and subjected to the treatment of UV-Ozone for 15 min. PEDOT:PSS in water (Clevios™ P VP AI 4083 from Heraeus) filtered by 0.45 µm polyvinylidene difluoride (PVDF) filter was then spun cast onto cleaned ITO substrates at 4000 rpm for 60 s and then baked at 130 °C for 15 min in air to give a thin film with a thickness of 40 nm. Then the perovskite film was coated on top of the PEDOT:PSS as previously mentioned. After cooling down, the [6,6]-phenyl C_61_ butyric acid methyl ester (PCBM) was spin-coated on top at 2000 rpm for 30 s from a PCBM solution in chloroform with a concentration of 13.3 mg mL^−1^ in a N_2_ filled glovebox. The film was heated at 80 °C for 15 min. After cooling down, a bathophenanthroline solution in ethanol with a concentration of 0.7 mg mL^−1^ was spin-coated on PCBM layer at 4000 rpm for 30 s. Then 70 nm aluminum was thermally evaporated as the metal electrode at a base pressure of 2 × 10^–6^ mbar. The active area was 0.13 cm^2^, controlled by a shadow mask.

Device characterization was carried out under AM 1.5 G irradiation with the intensity of 100 mW cm^−2^ (Oriel 91160, 300 W) calibrated by a NREL certified standard silicon cell in a nitrogen filled glovebox. Current density versus voltage (*J*–*V*) curves were recorded with a Keithley 2400 digital source meter. The scan rate is 50 mV s^−1^ and dwell time is 0.1 s. For oF1PEA, mF1PEA, pF1PEA based 2D OIHP solar cells, no preconditioning were performed while for PEA based 2D OIHP solar cell, light soaking for 10 min was performed before testing. External quantum efficiency (EQE) was detected under monochromatic illumination (Oriel Cornerstone 260 1/4 m monochromator equipped with Oriel 70613NS QTH lamp), and the calibration of the incident light was performed with a monocrystalline silicon diode (Model No.: Newport 71580).

### Optical characterization

The photoluminescence of OIHP thin films on glass slides was measured using a Horiba Scientific Fluorolog-3 spectrofluorometer. The excitation wavelength was 450 nm. The absorption of OIHP thin films on glass slides was obtained with a Shimadzu UV-2600 spectrophotometer.

All transient absorption experiments were conducted with a 45 fs, 4 mJ Coherent Libra with a 1 kHz repetition rate. Approximately 1.5 mJ of the 800 nm fundamental was focused into a 4 m long tube filled with argon gas to generate a visible continuum. The continuum pulse was then passed through an all-reflective 4 F setup, which was based on a 1200 g mm^−1^ grating and 20 cm focal length mirror. A motorized slit at the Fourier plane was used to filter the desired portion of the spectrum. The spectrally filtered pulses had 5 nm widths and 250 fs durations. Pulse energies were controlled with a rotational neutral density filter.

Continuum probe pulses were generated in a sapphire window and relayed to the sample with reflective optics. The spot size of the probe was adjusted to match the 200 µm spot size of the pump. The pump fluence was 2.9 × 10^13^ photons cm^−2^ (10 µJ cm^−2^) unless indicated otherwise below. Signal detection was accomplished with a CMOS array detector that was synchronized to the 1 kHz repetition rate of the laser system. In transient absorption experiments, the signals were averaged over 30 scans of the delay line and a total of 6000 differences were collected at each delay point.

### Surface morphology

For SEM and AFM imaging, films of the OIHPs were deposited on a PEDOT:PSS covered ITO substrate. SEM images were obtained by a Hitachi S-4700 cold cathode field emission SEM. AFM images were obtained by Asylum Research MFP3D Atomic Force Microscope.

### GIWAXS characterization

The GIWAXS patterns were collected at beamline 7.3.3 at the Advanced Light Source in Lawrence Berkeley National Laboratory^69^. The incident X-ray energy was 10 keV while the detector was a Pilatus 2 M. The patterns were corrected, axis-labeled, and colored as shown using Igor Pro and a modified version of the NIKA package^70^.

### Stability test

Devices of the OIHPs were fabricated following the same procedures for solar cell performance characterization except that Cu was used as the top electrode instead of Al. Un-encapsulated devices were kept in a desiccator with saturate K_2_CO_3_ to maintain a relative humidity around 45%.

### Single crystal growth

To synthesize the single crystals, 26.4 mg PbI_2_ and 28.6 mg PEAI (for oF1PEAI, mF1PEAI or pF1PEAI, 30.7 mg ammonium iodide was used) were dissolved in 57 wt% stabilized HI (Alfa Aesar) at 95 °C. 1.5 mL HI was needed to fully dissolve pF1PEAI, and for the rest, 1 mL was enough. This difference is likely due to the different formation energy of these perovskites. The solution was slowly cooled down at 1 °C h^−1^ to room temperature. The solids were filtered and washed with plenty of ethyl ether.

### Reporting Summary

Further information on experimental design is available in the Nature Research Reporting Summary linked to this article.

## Supplementary information

Supplementary Information

Source Data

## Data Availability

A reporting summary for this Article is available as a Supplementary Information file. The source data underlying Supplementary Figure 3 and Supplementary Table 1 are provided as a Source Data file. The single crystal results are available in The Cambridge Crystallographic Data Center: oF1PEA_2_PbI_4_: 1893383 [10.5517/ccdc.csd.cc21k6wq]; mF1PEA_2_PbI_4_: 1893384 [10.5517/ccdc.csd.cc21k6xr]; pF1PEA_2_PbI_4_: 1893385 [10.5517/ccdc.csd.cc21k6ys]; idealized supercell of oF1PEA_2_PbI_4_: 1893474 [10.5517/ccdc.csd.cc21k9tr]; idealized supercell of mF1PEA_2_PbI_4_: 1893475 [10.5517/ccdc.csd.cc21k9vs]. All relevant data are available from the authors.
