# Supplementary material for: Synthetic control over orientational degeneracy of spacer cations enhances solar cell efficiency in two-dimensional perovskites
Source: Nat Commun. 2019 Mar 20;10:1276. doi: 10.1038/s41467-019-08980-x (PMC6427015; doi:10.1038/s41467-019-08980-x)
Supplement: Supplementary file 1 — Supplementary Information [file 41467_2019_8980_MOESM1_ESM.pdf]

Supplementary Information

Synthetic Control over Orientational Degeneracy of Spacer Cations Enhances Solar Cell Efficiency in Two-Dimensional Perovskites

Hu *et. al.*

Supplementary Figures

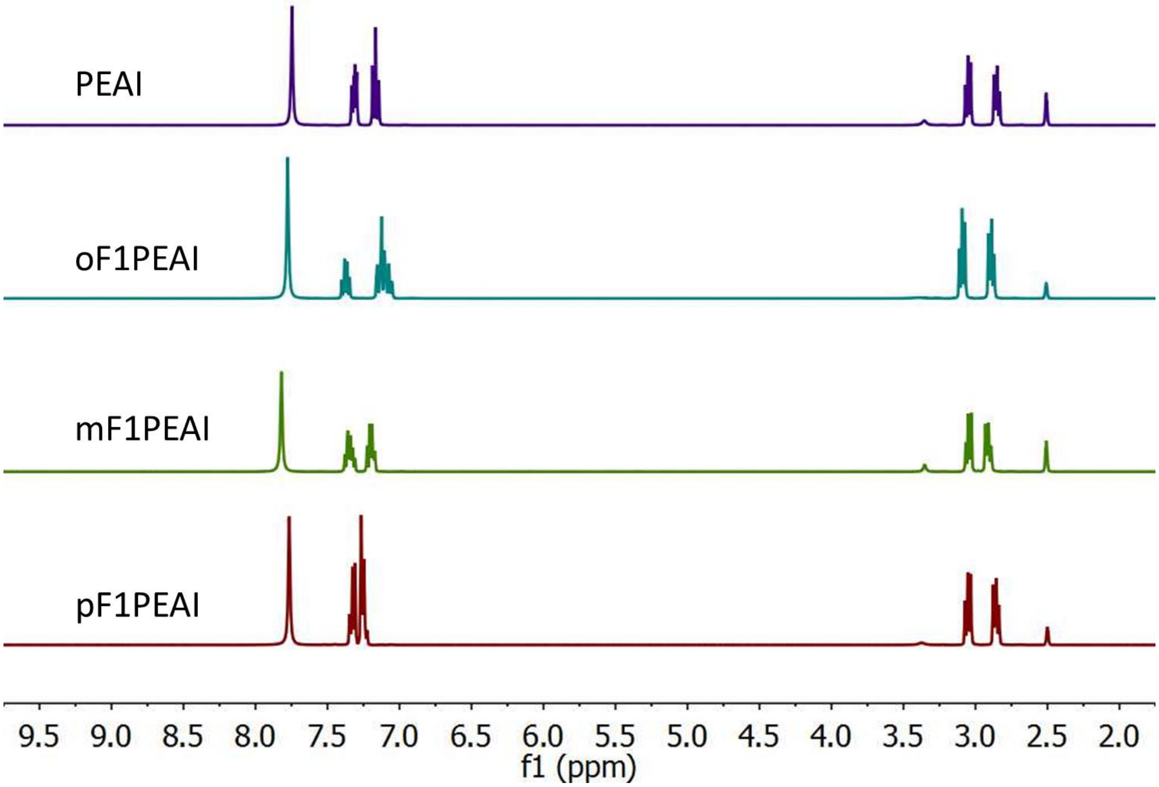

Supplementary Figure 1 | Characterization of synthesized organic cations. Structures and ^{1}H NMR (dimethyl sulfoxide-d_6, 400 MHz) of PEA based ammonium.

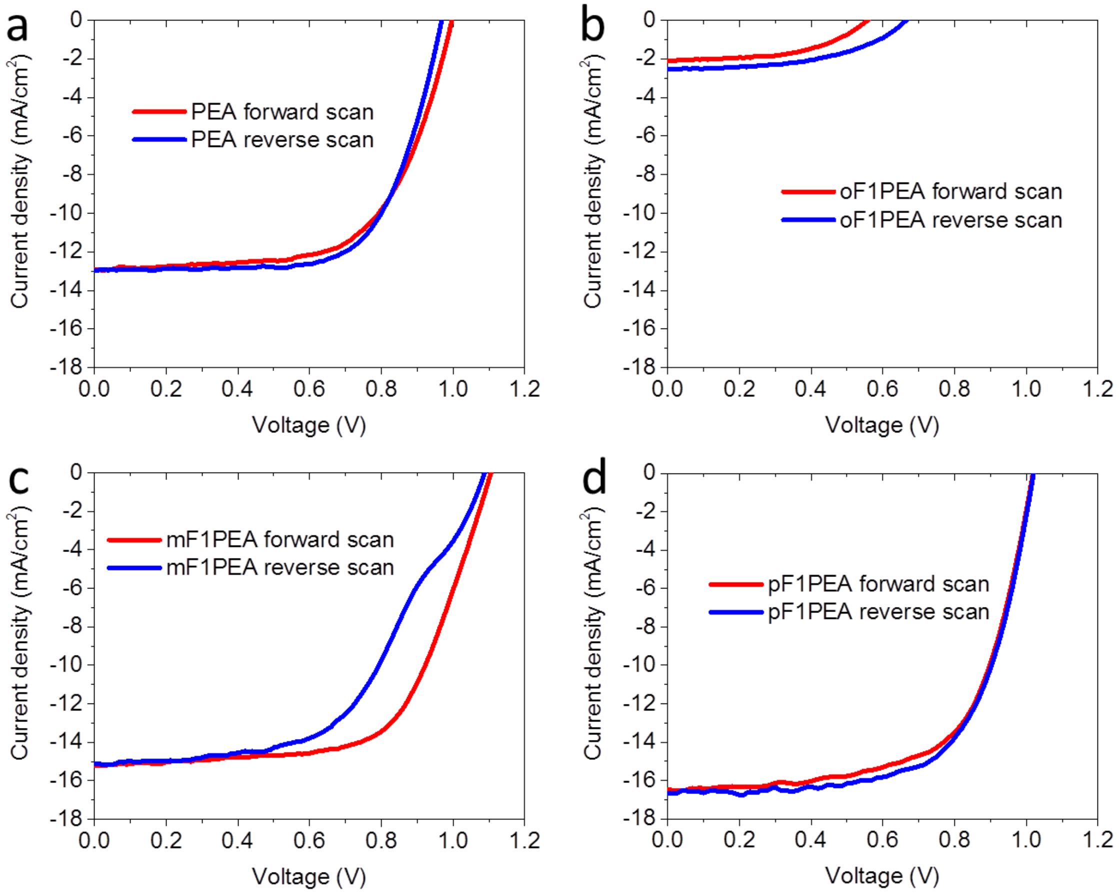

Supplementary Figure 2 | Current-density-voltage (*J*-*V*) curves under an AM.1.5G solar simulator. *J*-*V* curves of 2D OIHP films based on PEA (a), oF1PEA (b), mF1PEA (c) and pF1PEA (d).

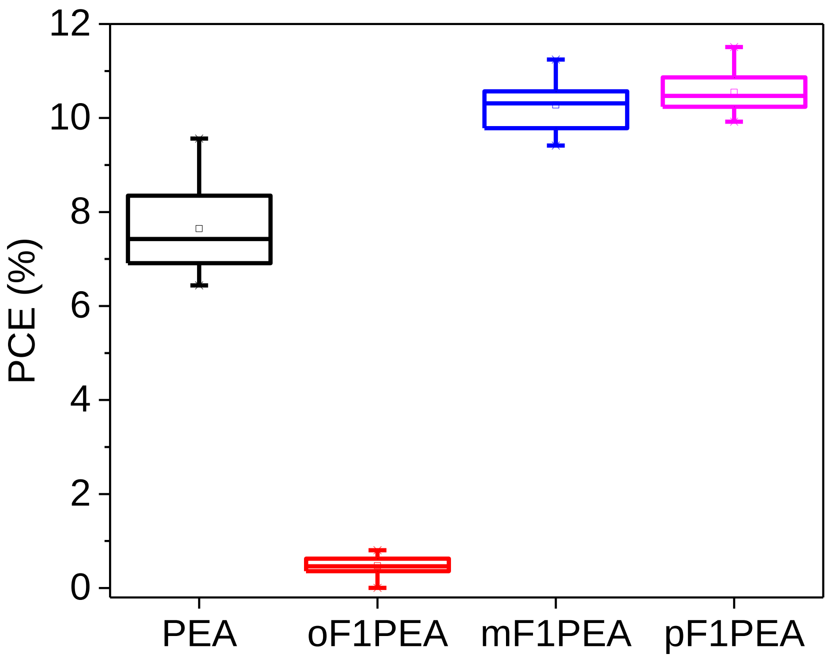

Supplementary Figure 3 | Statistical result of device efficiency. Efficiency box chart of ~ 40 2D OIHP solar cells based on different cations. Maximum, minimum, third quartile, first quartile, median and average are provided. Source data are provided as a Source Data file.

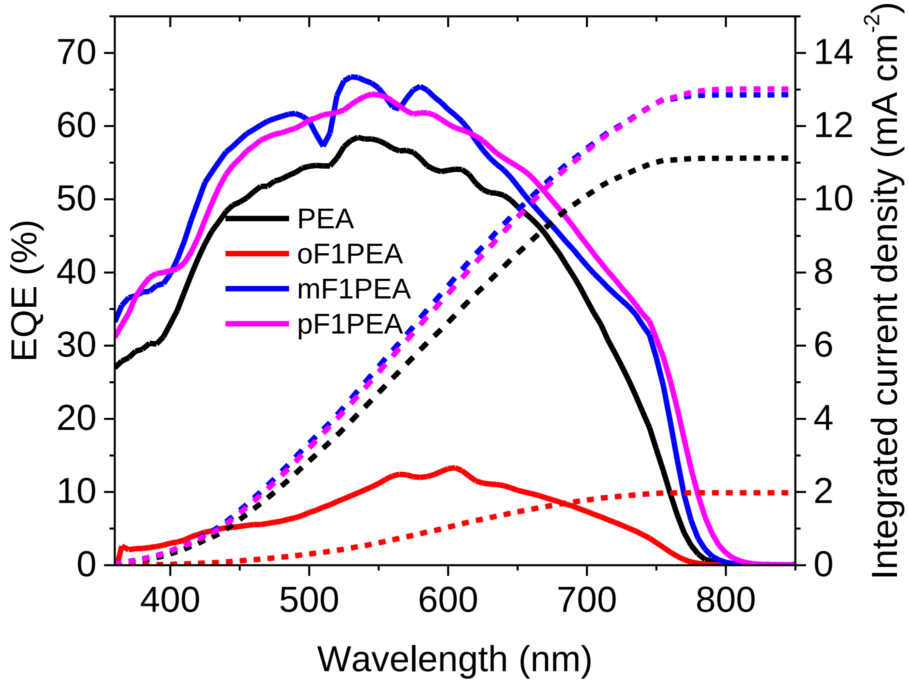

Supplementary Figure 4 | external quantum efficiency (EQE) and integrated current density as a function of wavelength

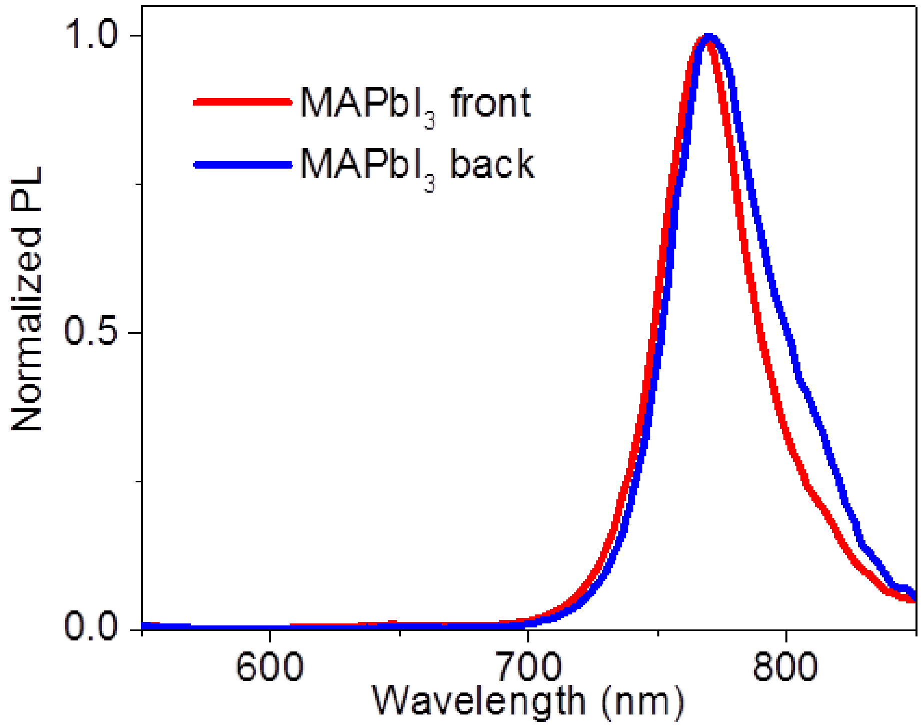

Supplementary Figure 5 | PL of 3D perovskite ($MAPbI_3$). Red curve is excited from front (air) sides of the film and blue curve is excited from back (glass) sides of the film.

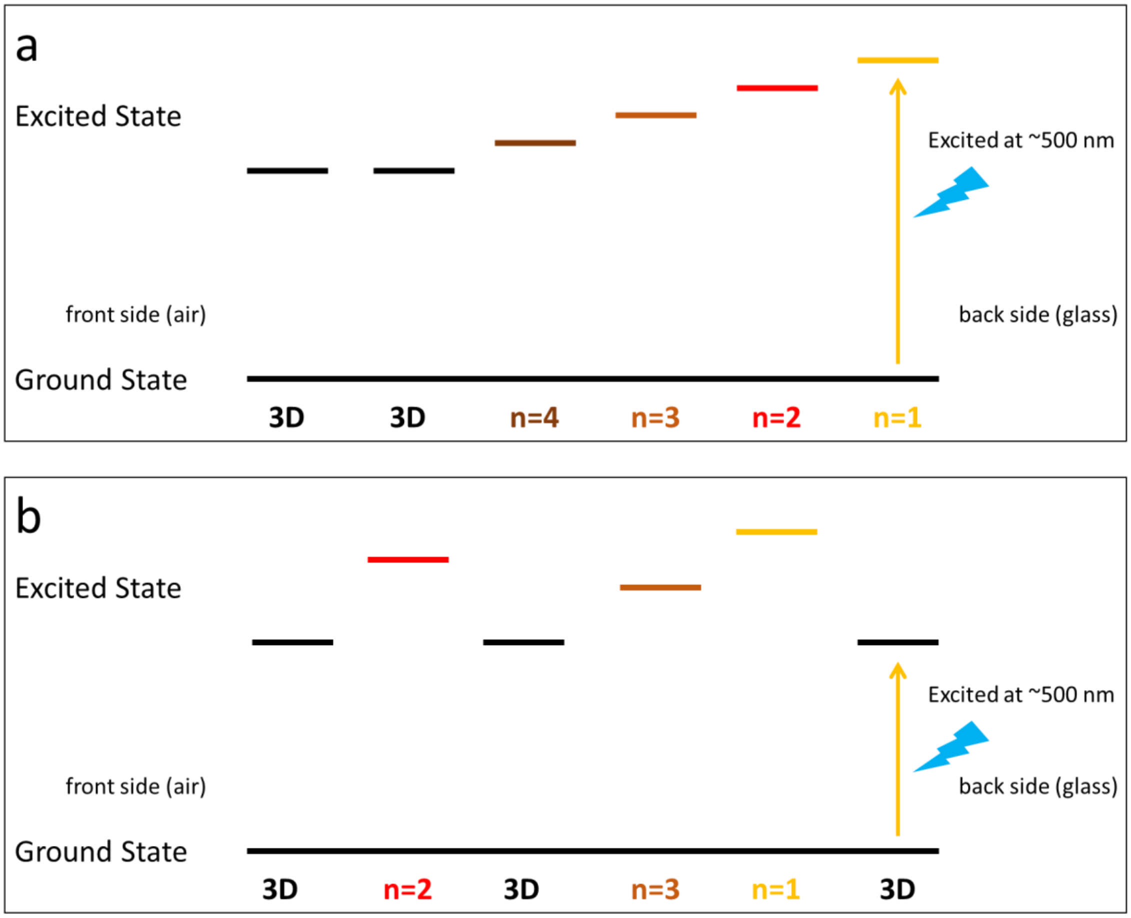

Supplementary Figure 6 | Different phase distributions in 2D OIHP films. (a) ordered phase distribution with a greater amount of smaller *n* phases at the ITO side and larger amount of large *n* and 3D phases near the Al side (i.e., the air side). (b) disordered phased distribution in the film, which would cause charge trapping in the film.

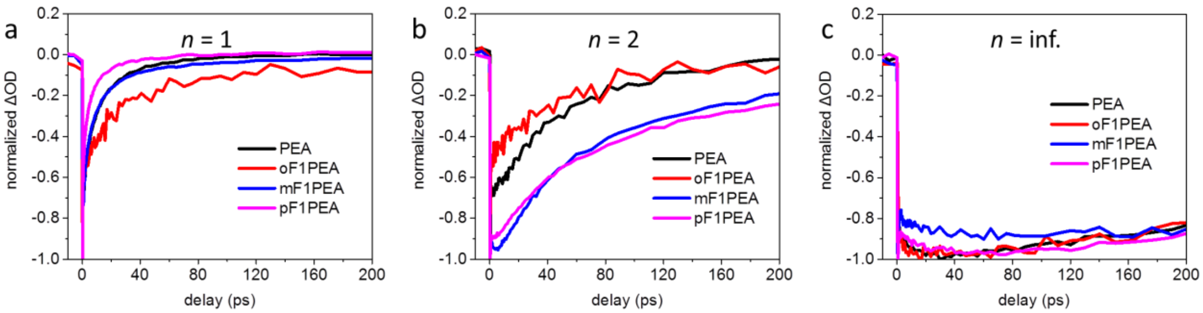

Supplementary Figure 7 | Transient absorption signal dynamics under the absorption wavelength of $n = 1$ (a), $n = 2$ (b) and $n = \infty$ (c).

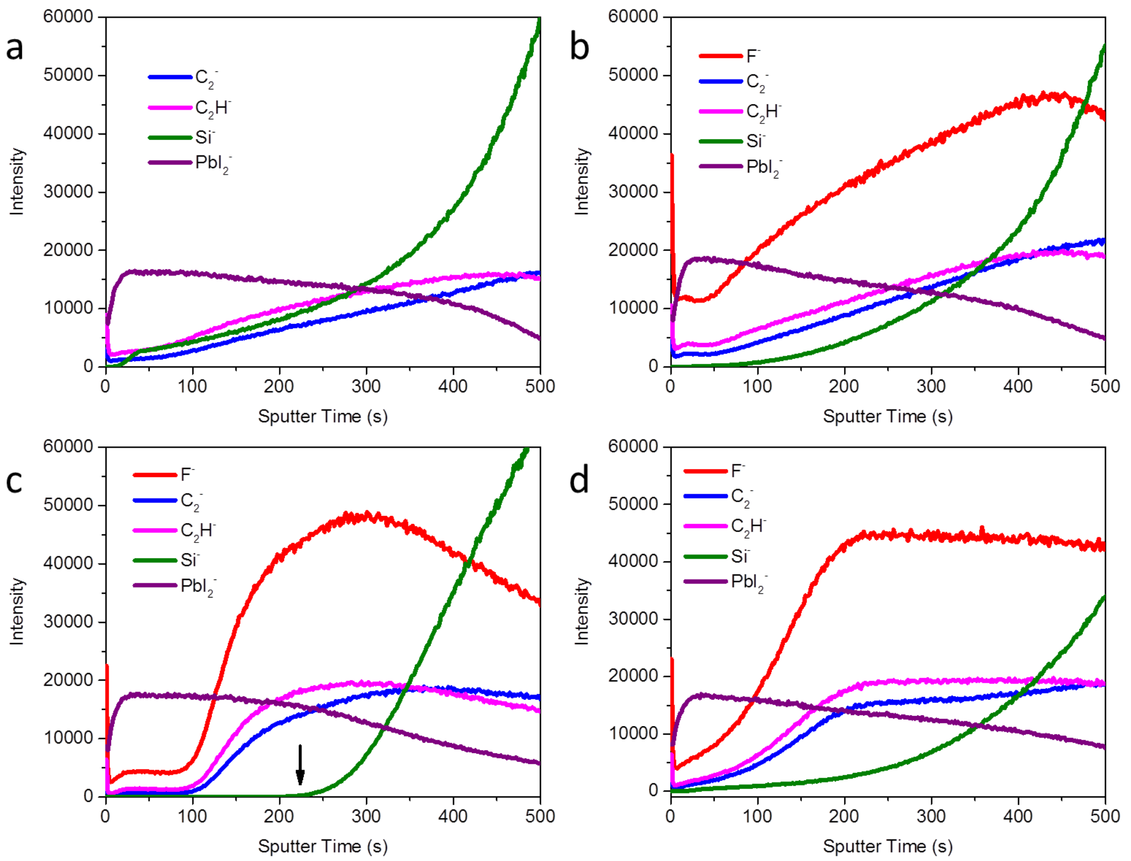

Supplementary Figure 8 | ToF-SIMS result of 2D OIHPs (n = 4) based on PEA (a), oF1PEA (b), mF1PEA (c) and pF1PEA (d). The arrow in (c) indicates the time substrate appears.

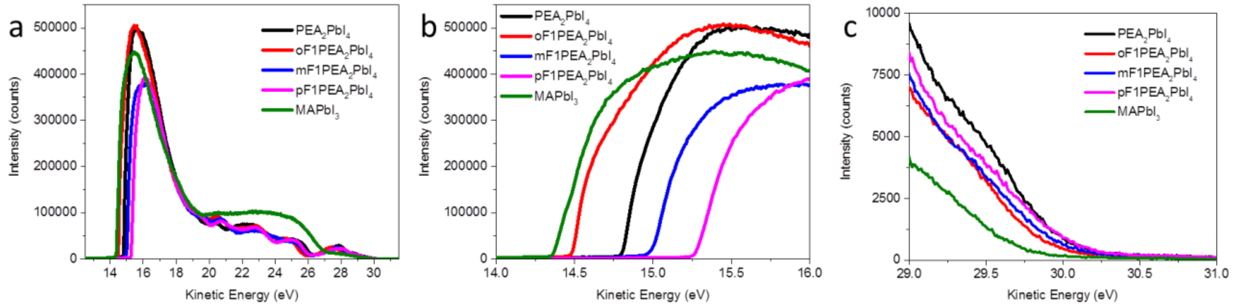

Supplementary Figure 9 | UPS result of different 2D OIHPs of n = 1 and 3D OIHP. (a) full spectra, (b) low kinetic energy edge region, (c) high kinetic energy edge region.

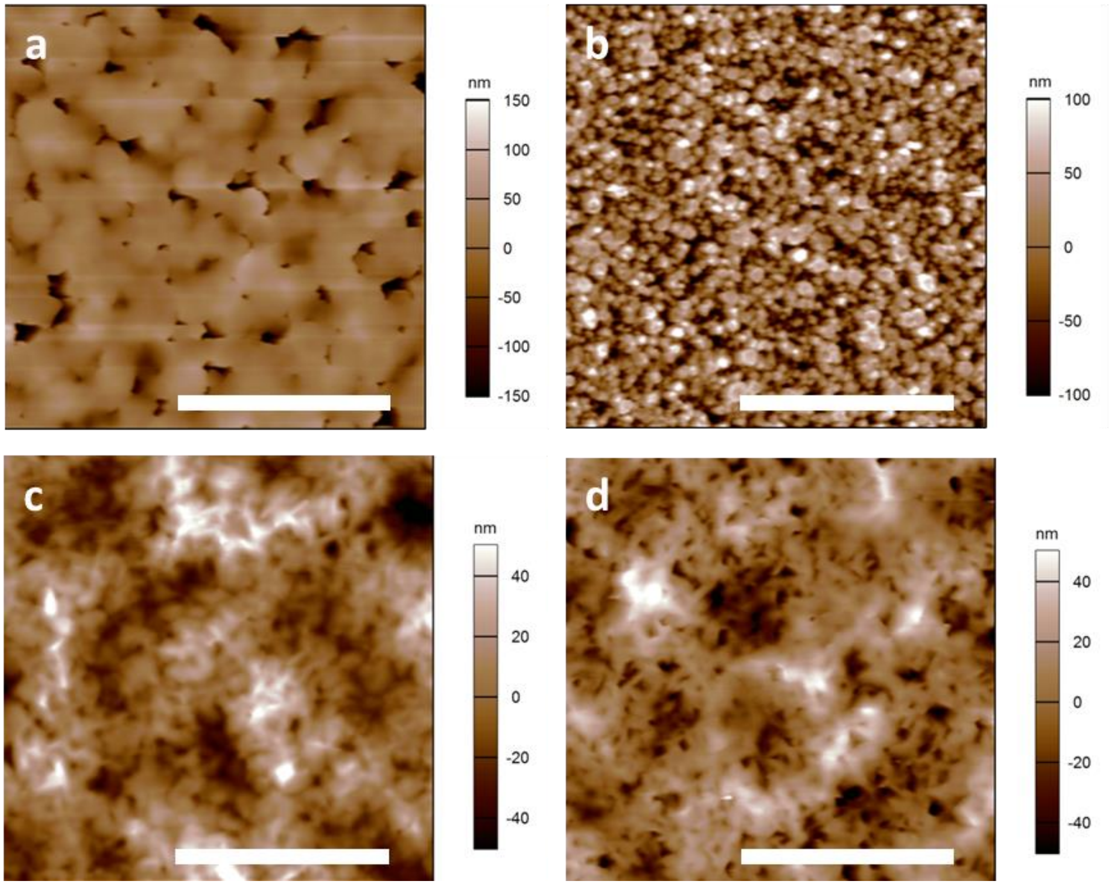

Supplementary Figure 10 | Surface morphology of 2D OIHP films. AFM images of 2D OIHP films based on (a) PEA, (b) oF1PEA, (c) mF1PEA and (d) pF1PEA. The roughness of each film is 34.6 nm, 44.6 nm, 18.9 nm and 17.7 nm, respectively. Scale bar is 10 µm.

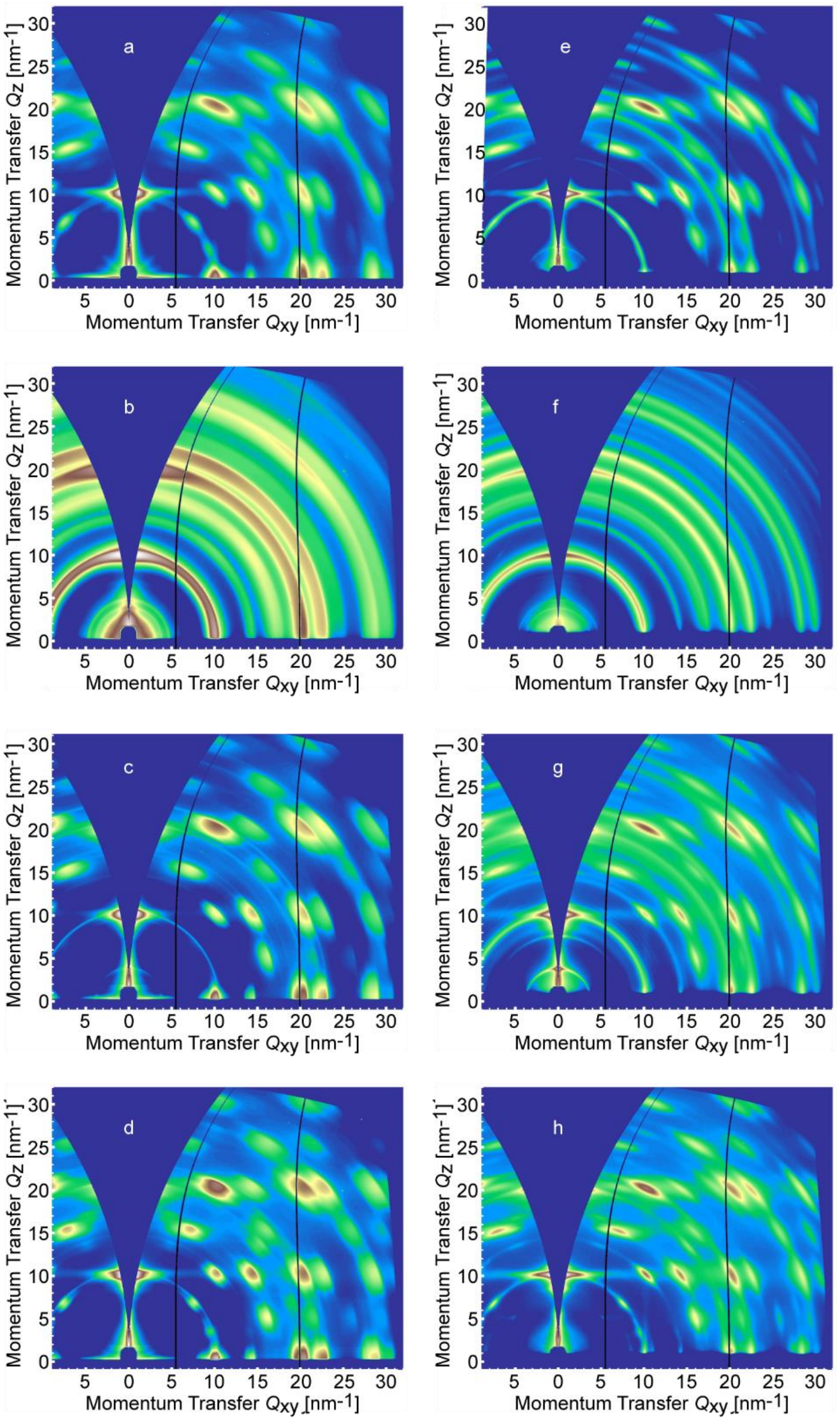

Supplementary Figure 11 | GIWAXS patterns of 2D OIHP films with PEA and fluorinated PEAs under different incident angles: (a, e) PEA, (b, f) oF1PEA, (c, g) mF1PEA and (d, h) pF1PEA films probing at two different X-ray incident angles; left column (a-d) shows patterns acquired at incident angle = 0.18°, probing a few namometers of the perovskite films from the front (air) interface, and right column (e-h) shows patterns acquired at incident angle = 1°, probing more into the bulk of the films.

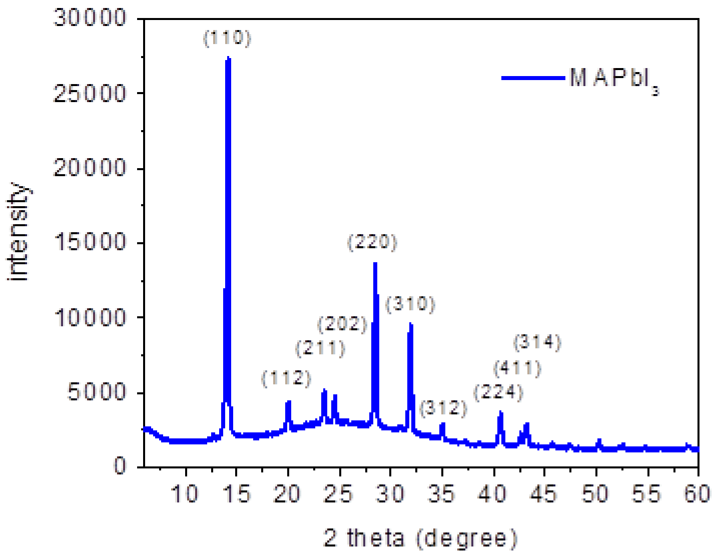

Supplementary Figure 12 | XRD pattern of 3D perovskite: XRD pattern of $MAPbI_3$. Peak indexes are labelled.

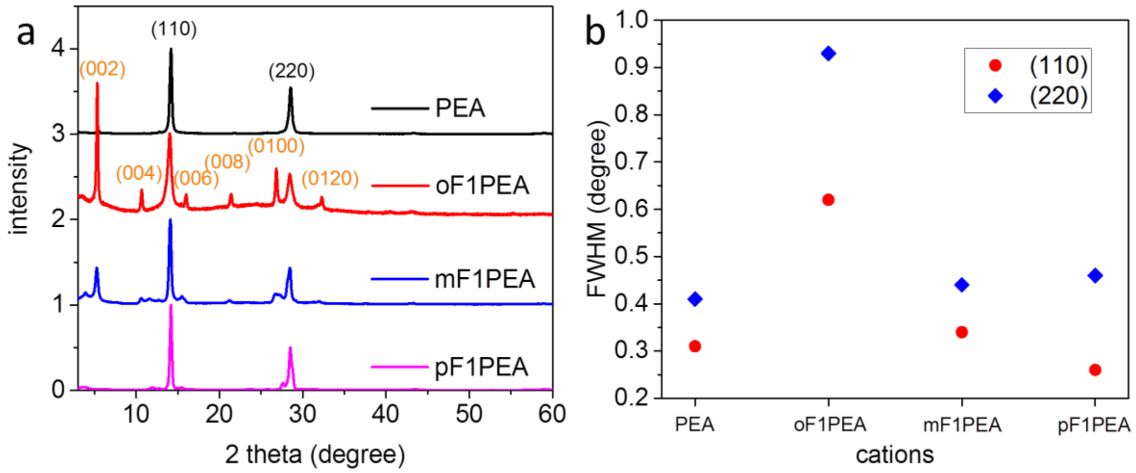

Supplementary Figure 13 | XRD patterns of 2D OIHP films with PEA and fluorinated PEAs: **a,** XRD of different 2D OIHP films; **b,** FWHM of peak (110) and (220) in different films. The orange (*00l*) peak indexes are corresponding to the n = 1 phase; (110) and (220) peaks can exist in both 2D and 3D perovskite phases.

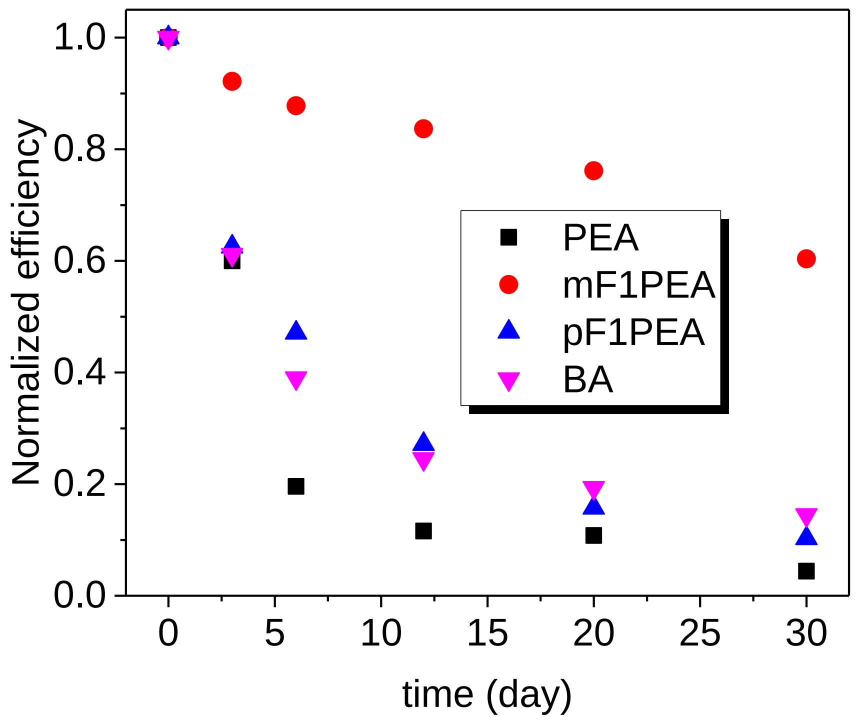

Supplementary Figure 14 | Stability test: stability of 2D OIHPs based on different organic cations including PEA, F1PEA and BA under ambient condition (RH=45%) for 30 days.

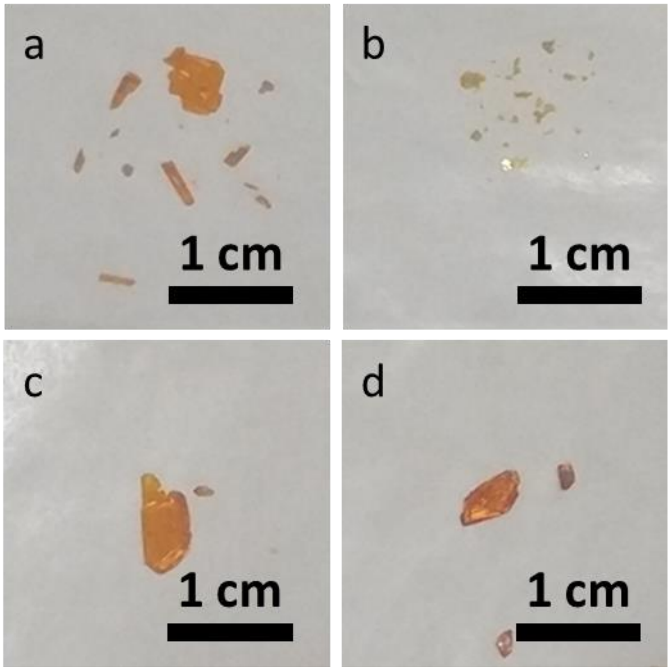

Supplementary Figure 15 | Single crystals of 2D OIHPs (*n* = 1) with different PEA cations. (a) PEA_2PbI_4, (b) $oF1PEA_2PbI_4$, (c) $mF1PEA_2PbI_4$, (d) $pF1PEA_2PbI_4$.

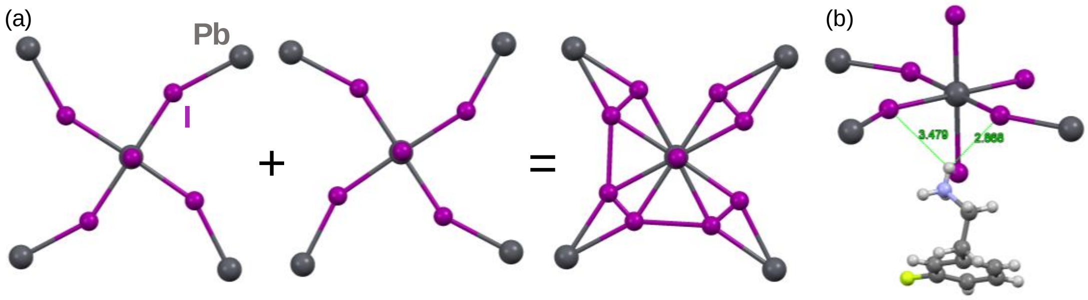

Supplementary Figure 16 | Disorder behavior of mF1PEA$_2$PbI$_4$ crystal structure: (**a**) The two isolated [PbI$_6$] octahedra that comprise the average (right) inorganic unit in mF1PEA$_2$PbI$_4$. (**b**) A single inorganic unit with the gauche-conformation ammonium group pointing towards the "puckered out" I-Pb-I bond with realistic interatomic distances.

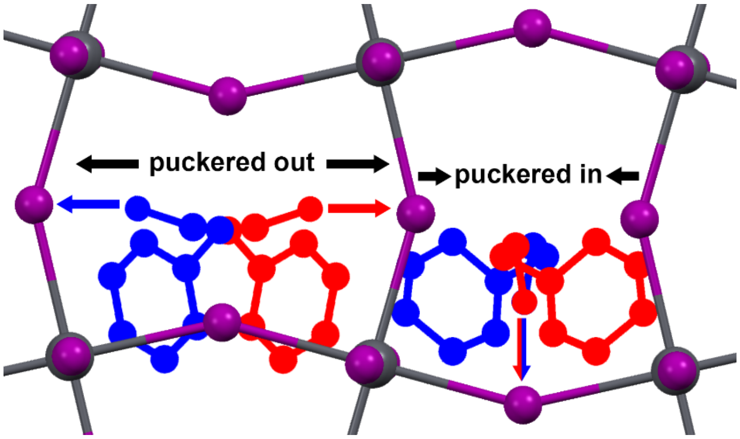

Supplementary Figure 17 | When the aromatic moiety is directed towards the puckered out I-Pb-I bond, two possible ammonium orientations exist. When the aromatics are directed towards the puckered in geometry, only one possible ammonium orientation exists.

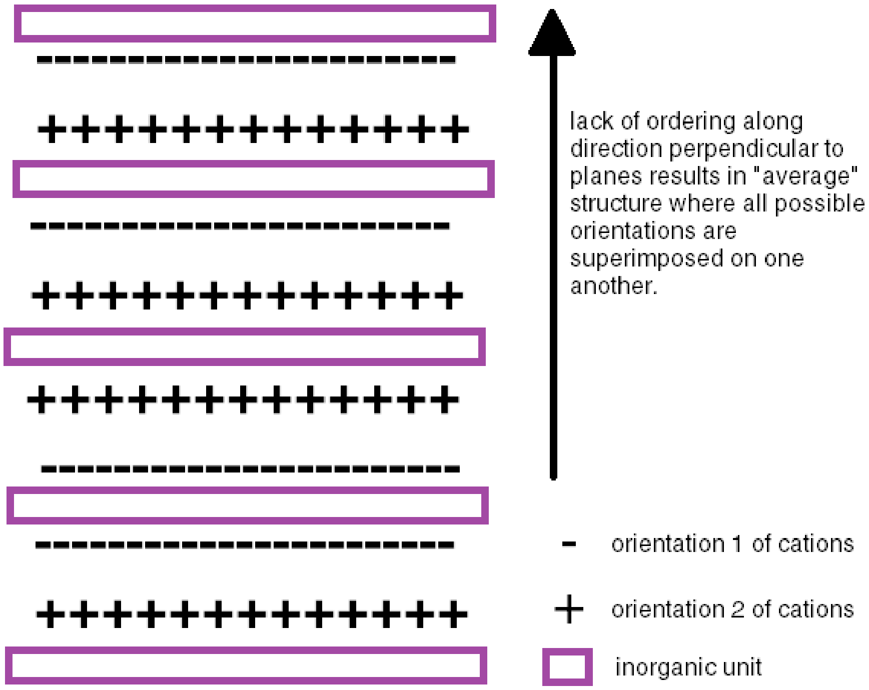

Supplementary Figure 18 | Cartoon showing the disorder of oF1PEA cations in oF1PEA$_2$PbI$_4$. In ordered structure, "+|+-|-+|+-|-" or "+|-+|-+|-" type structure is formed. However, we do not observe this and instead see the average structure as shown, indicating the packing is random.

Supplementary Tables

Supplementary Table 1 | Photovoltaic device performance of 2D OIHP based on PEA and fluorinated PEAs. Source data are provided as a Source Data file.

Cation	scan direction	J_{sc} (mA cm^{-2})	V_{oc} (V)	FF (%)	Efficiency *(η)* (%)
PEA	forward	13.02±0.74	0.971±0.063	60.6±3.7	7.67±0.90
	reverse	12.99±0.74	0.949±0.044	64.4±4.3	7.95±0.88
oF1PEA	forward	2.41±1.41	0.570±0.254	44.4±8.7	0.50±0.16
	reverse	2.71±1.70	0.660±0.236	47.6±8.8	0.73±0.31
mF1PEA	forward	15.07±1.00	1.075±0.017	62.9±3.1	10.17±0.52
	reverse	14.94±0.95	1.077±0.017	58.42±3.1	9.40±0.68
pF1PEA	forward	16.37±0.89	1.025±0.032	63.0±2.9	10.55±0.38
	reverse	16.63±0.88	1.034±0.031	63.4±3.8	10.89±0.55

Supplementary Table 2 | Optimization of annealing temperature for 2D OIHP solar cell fabrication.

Cation	Annealing Temperature (°C)	Scan Direction	J_{sc} (mA cm^{-2})	V_{oc} (V)	FF (%)	η (%)
PEA	33	forward	12.76	0.891	57.0	6.50
		reverse	12.79	0.888	64.3	7.33
	40	forward	12.74	0.962	62.2	7.64
		reverse	12.69	0.947	66.9	8.05
	45	forward	12.27	0.937	59.8	6.86
		reverse	12.23	0.932	61.2	6.98
	60	forward	13.06	1.124	41.6	6.11
		reverse	13.23	1.066	41.1	5.78
oF1PEA	32	forward	5.17	0.323	37.8	0.64
		reverse	6.07	0.474	45.3	1.30
	40	forward	1.89	0.519	49.5	0.50
		reverse	2.17	0.533	75.7	0.69
	50	forward	0.94	0.440	48.6	0.21
		reverse	1.30	0.598	43.2	0.33
	60	forward	1.80	0.378	49.8	0.33
		reverse	1.84	0.450	52.9	0.42
mF1PEA	32	forward	13.41	1.037	63.2	8.79

		reverse	13.53	1.030	57.7	8.03
	40	forward	14.63	1.108	62.6	10.17
		reverse	14.56	1.097	52.4	8.38
	50	forward	14.13	1.045	57.0	8.40
		reverse	13.98	1.064	49.4	7.34
	60	forward	13.08	1.044	53.6	7.31
		reverse	12.98	1.086	47.3	6.67
pF1PEA	33	forward	10.14	0.353	41.7	1.49
		reverse	10.77	0.359	44.5	1.73
	40	forward	13.37	0.673	52.7	4.76
		reverse	13.80	0.688	53.4	5.09
	50	forward	13.94	0.991	64.5	8.92
		reverse	14.24	0.986	66.3	9.31
	60	forward	15.16	1.106	62.1	10.41
		reverse	15.48	1.108	66.3	11.38
	65	forward	16.61	1.012	63.7	10.70
		reverse	16.76	1.015	65.2	11.08
	80	forward	12.39	1.037	55.0	7.06
		reverse	12.45	1.066	59.0	7.82

Supplementary Table 3 | Thicknesses of 2D OIHP layer in the solar cells based on PEA, oF1PEA, mF1PEA and pF1PEA

**Cation**	PEA	oF1PEA	mF1PEA	pF1PEA
**Thickness (nm)**	326	340	320	359

Supplementary Table 4 | Summary of energy levels obtained from UPS

sample	low KE edge (eV)	high KE edge (eV)	work function (eV)	VB (eV)	E_g (eV)	CB (eV)
PEA_2PbI_4	14.8	30.0	4.8	-6.0	2.4	-3.6
$oF1PEA_2PbI_4$	14.5	29.9	4.5	-5.8	2.5	-3.4
$mF1PEA_2PbI_4$	15.0	30.0	5.0	-6.2	2.4	-3.7
$pF1PEA_2PbI_4$	15.3	30.1	5.3	-6.4	2.4	-4.0
$MAPbI_3$	14.4	29.8	4.4	-5.8	1.5	-4.2

Supplementary Table 5 | pF1PEA, mF1PEA, and oF1PEA Atomic Positions and Thermal Parameters†

Atom	*x*	*y*	*z*	$U_{iso}{}^{*}/U_{eq}$ (Å^2)^a	Occ. (<1)
**$pF1PEA_2PbI_4$**					
Pb1	0	0	0	0.02830 (8)	
I1	−0.00020 (2)	−0.31108 (3)	0.19089 (3)	0.04120 (9)	
I2	0.19457 (2)	−0.01738 (3)	0.06893 (4)	0.04012 (9)	
N1	0.1612 (2)	−0.0746 (5)	0.4671 (4)	0.0457 (9)	
C1	0.1955 (3)	0.0378 (6)	0.5894 (6)	0.0525 (12)	
C2	0.2823 (4)	−0.0041 (7)	0.6497 (7)	0.0606 (16)	
C3	0.3367 (3)	−0.0010 (5)	0.5293 (6)	0.0459 (12)	
C4	0.3640 (3)	−0.1378 (7)	0.4734 (6)	0.0540 (12)	
C5	0.4130 (3)	−0.1381 (8)	0.3595 (8)	0.0662 (16)	
C6	0.4339 (3)	0.0027 (7)	0.3038 (7)	0.0606 (16)	
C7	0.4085 (3)	0.1414 (7)	0.3565 (7)	0.0600 (14)	
C8	0.3597 (3)	0.1380 (7)	0.4685 (6)	0.0543 (12)	
F1	0.4816 (3)	0.0039 (5)	0.1913 (6)	0.0989 (16)	
**$mF1PEA_2PbI_4$**					
Pb1	0	0.48960 (4)	0.250000	0.04751 (11)	
I1	−0.09615 (2)	0.49011 (6)	0.19080 (3)	0.04871 (11)	
I2	−0.00096 (2)	0.62674 (11)	0.00054 (5)	0.04087 (16)	0.5
I3	−0.00056 (2)	−0.00921 (9)	0.18551 (6)	0.04325 (17)	0.5
N1A	0.0804 (3)	0.9870 (13)	0.1328 (8)	0.0498 (16)	0.509(7)
C1A	0.0976 (6)	0.998 (3)	0.0331 (13)	0.052 (3)	0.509(7)
N1B	0.0798 (3)	0.8205 (14)	0.0559 (8)	0.0498 (16)	0.491(7)
C1B	0.0971 (7)	1.045 (3)	0.0734 (13)	0.052 (3)	0.491(7)
C2	0.1395 (2)	1.0595 (13)	0.0651 (6)	0.0729 (19)	
C3	0.16650 (17)	0.9093 (11)	0.1446 (6)	0.0567 (15)	
C4	0.1780 (2)	0.9566 (13)	0.2559 (7)	0.0731 (19)	
C5	0.2015 (2)	0.8122 (18)	0.3262 (7)	0.087 (3)	
C6	0.2152 (2)	0.6259 (17)	0.2910 (10)	0.100 (3)	
C7	0.2035 (2)	0.5773 (16)	0.1787 (9)	0.094 (3)	
C8	0.1800 (2)	0.7203 (13)	0.1068 (7)	0.079 (2)	
F1	0.21242 (17)	0.8648 (16)	0.4369 (5)	0.162 (3)	
**$oF1PEA_2PbI_4$**					
Pb1	½	1	½	0.04227 (10)	
Pb2	1	½	½	0.04236 (10)	
I1	0.68475 (5)	0.68475 (5)	0.50011 (3)	0.05014 (13)	
I2	0.81215 (5)	1.18741 (5)	0.50065 (3)	0.05375 (13)	
I3	0.93898 (6)	0.44789 (6)	0.69281 (3)	0.05749 (13)	

I4	0.45316 (6)	0.96615 (6)	0.69279 (3)	0.05805 (13)	
N2	0.5433 (7)	0.3753 (7)	0.6649 (4)	0.0599 (15)	
F1A	1.110 (3)	0.696 (3)	0.882 (2)	0.100 (6)	0.506(8)
F2A	0.589 (11)	0.192 (4)	0.866 (7)	0.150 (10)	0.506(8)
N1A	1.0531 (15)	1.0502 (14)	0.6579 (7)	0.059 (3)	0.506(8)
C1A	0.949 (3)	0.937 (3)	0.7079 (16)	0.076 (4)*	0.506(8)
C2A	0.898 (4)	0.896 (4)	0.7917 (19)	0.120 (7)*	0.506(8)
C3A	1.0029 (15)	0.9400 (15)	0.8454 (8)	0.071 (4)*	0.506(8)
C4A	1.0951 (17)	0.8309 (13)	0.8840 (9)	0.086 (6)*	0.506(8)
C5A	1.1901 (17)	0.8744 (18)	0.9375 (9)	0.106 (7)*	0.506(8)
C6A	1.101 (2)	1.1360 (15)	0.9137 (11)	0.124 (9)*	0.506(8)
C7A	1.193 (2)	1.027 (2)	0.9524 (10)	0.117 (9)*	0.506(8)
C8A	1.0058 (17)	1.0925 (14)	0.8603 (9)	0.091 (6)*	0.506(8)
C9A	0.409 (3)	0.461 (3)	0.7012 (16)	0.068 (3)*	0.506(8)
C10A	0.371 (3)	0.410 (3)	0.7913 (13)	0.084 (5)*	0.506(8)
C11A	0.4908 (15)	0.4488 (15)	0.8432 (8)	0.070 (4)*	0.506(8)
C12A	0.5870 (17)	0.3394 (13)	0.8784 (10)	0.090 (6)*	0.506(8)
C13A	0.6905 (17)	0.3828 (18)	0.9277 (9)	0.107 (7)*	0.506(8)
C14A	0.6979 (19)	0.536 (2)	0.9418 (10)	0.115 (9)*	0.506(8)
C15A	0.602 (2)	0.6449 (15)	0.9065 (11)	0.125 (10)*	0.506(8)
C16A	0.4982 (17)	0.6015 (14)	0.8572 (9)	0.086 (6)*	0.506(8)
F1B	1.102 (6)	0.709 (6)	0.853 (3)	0.21 (2)	0.494(8)
F2B	0.613 (11)	0.247 (5)	0.857 (7)	0.150 (10)	0.494(8)
N1B	0.8729 (17)	0.8722 (16)	0.6565 (9)	0.070 (4)	0.494(8)
C1B	0.987 (3)	0.917 (3)	0.7032 (17)	0.076 (4)*	0.494(8)
C2B	0.956 (4)	0.952 (4)	0.7927 (19)	0.120 (7)*	0.494(8)
C3B	0.8850 (16)	0.8189 (15)	0.8464 (9)	0.077 (5)*	0.494(8)
C4B	0.9715 (13)	0.7056 (18)	0.8859 (10)	0.081 (6)*	0.494(8)
C5B	0.898 (2)	0.5846 (17)	0.9383 (10)	0.113 (8)*	0.494(8)
C6B	0.738 (2)	0.577 (2)	0.9513 (12)	0.123 (10)*	0.494(8)
C7B	0.6511 (14)	0.690 (2)	0.9118 (12)	0.133 (11)*	0.494(8)
C8B	0.7248 (15)	0.8113 (18)	0.8593 (10)	0.089 (6)*	0.494(8)
C9B	0.439 (3)	0.491 (3)	0.6998 (16)	0.068 (3)*	0.494(8)
C10B	0.448 (3)	0.484 (3)	0.7905 (12)	0.080 (5)*	0.494(8)
C11B	0.3832 (14)	0.3419 (14)	0.8422 (8)	0.063 (4)*	0.494(8)
C12B	0.4758 (13)	0.2294 (17)	0.8778 (10)	0.086 (6)*	0.494(8)
C13B	0.409 (2)	0.1004 (16)	0.9266 (10)	0.109 (8)*	0.494(8)
C14B	0.249 (2)	0.0840 (18)	0.9399 (11)	0.120 (9)*	0.494(8)
C15B	0.1566 (13)	0.197 (2)	0.9043 (12)	0.149 (13)*	0.494(8)

C16B	0.2235 (14)	0.3255 (17)	0.8555 (10)	0.087 (6)*	0.494(8)

†Data from the solved crystal structures (not idealized supercells). [a] U_{eq} is defined as one-third of the trace of the orthogonalized U_{ij} tensor.

Supplementary Table 6 | pF1PEA, mF1PEA, and oF1PEA Experimental Crystallographic Parameters†

Compound	$pF1PEA_2PbI_4$	$mF1PEA_2PbI_4$	$oF1PEA_2PbI_4$
Formula	$C_{16}H_{22}F_2I_4N_2Pb$	$C_{16}H_{22}F_2I_4N_2Pb$	$C_{16}H_{22}F_2I_4N_2Pb$
Crystal System	Monoclinic	Monoclinic	Triclinic
Space Group	$P2_1/c$	$C2$/c	$P\bar{1}$
a (Å)	16.723(2)	34.6431(17)	8.7354(2)
b (Å)	8.6332(12)	6.1150(3)	8.7379(2)
c (Å)	8.8000(12)	12.2922(6)	16.9013(5)
α (°)	90	90	82.218(1)
β (°)	98.781(4)	103.930(2)	82.914(1)
ɣ (°)	90	90	89.596(1)
V (Å^3)	1255.6(3)	2527.4(2)	1268.36(6)
Z	2	4	2
Crystal dimensions (mm^3)	0.08 × 0.04 × 0.002	0.12 × 0.09 × 0.03	0.08 × 0.07 × 0.02
θ range (º)	2.5 – 26.5	2.4 – 26.4	3.2 – 28.9
μ (mm^{-1})	11.65	11.58	11.54
Temperature (K)	300(2)	302(2)	300(2)
Measured Reflections	20392	19782	25944

Independent Reflections	2594	2613	7843
Reflections with $I > 2\sigma(I)$	2330	1983	4989
R_{int}	0.040	0.042	0.068
R_1 $(F)^a$	0.021	0.027	0.043
wR_2^b	0.058	0.070	0.114
Parameters	115	130	184
Goodness-of-fit on F^2	1.05	1.05	0.96
largest diff. peak and hole (e Å^{-3})	2.37 and -0.85	0.67 and -2.04	2.26 and -2.26

†Data from the solved crystal structures (not idealized supercells). $^aR_1 = \Sigma||F_o| - |F_c||/ \Sigma|F_o|$. $^bwR_2 = [\Sigma[w(F_o^2 - F_c^2)^2]/ \Sigma[w(F_o^2)^2]]^{1/2}$.

Supplementary Table 7 | Summary of energetics calculated using density functional theory*

Organic Cation	E_{A2PbI4} (kJ mol^{-1})	E_{AI} (kJ mol^{-1})	ΔH_f^{0K} (kJ mol^{-1})	$\Delta\Delta H_f^{0K}$ (kJ mol^{-1}) (relative to *pF1PEA$_2$PbI$_4$*)
p-$FC_6H_4(CH_2)_2NH_3^+$	-10813	-20778	1006	0
m-$FC_6H_4(CH_2)_2NH_3^+$	-10814	-20681	1107	101
o-$FC_6H_4(CH_2)_2NH_3^+$	-10807	-20155	1617	611
$C_6H_5(CH_2)_2NH_3^+$	-10965	-20873	1214	209

* $\Delta H_f^{0K} \approx E_{A2PbI4} - (2E_{AI} + E_{PbI2})$. E_{PbI2} = -158.0 kJ mol^{-1}

Supplementary Table 8 | Bond angle and length for 2D OIHP single crystals

Perovskite	Absorption peak (nm)*	Pb-I-Pb bond angle (°)	Pb-I bond length (Å)
**PEA_2PbI_4**	517	152.98	3.1713
**$oF1PEA_2PbI_4$**	506	151.09	3.1715
**$mF1PEA_2PbI_4$**	510	152.15	3.1945
**$pF1PEA_2PbI_4$**	518	152.94	3.1693

* absorption peak from UV-vis spectrum of thin films

Supplementary Notes

Supplementary Note 1

We conducted EQE measurement on each 2D perovskite solar cells without light bias. The integrated current density from EQE is lower than the current density achieved from *J-V* cruves under one sun conduction. This can be caused by the existence of multiple phases in our 2D perovskite films. For a standard EQE measurement without light bias, the large band gap components are not excited, which might cause a large resistance in the cell and lower the current measured. Such phenomenon has also been seen in tandem solar cells[1-4] and Cu_2ZnSnS_4 solar cells.[5]

Supplementary Note 2

The dynamics of n = 1, 2 and 3D phases for each 2D OIHP sample show a clear decay of n = 1 for all four 2D OIHP films (**Supplementary Figure 7a**), however, there is a clear increase of the absorption intensity at early stage (< 10 ps) in the decay curve of n = 2 for mF1PEA and pF1PEA 2D OIHP films (**Supplementary Figure 7b**). For PEA based 2D OIHP film, though there is no clear increase of the absorption intensity for n = 2, the decay of the absorption intensity (n = 2) for PEA is much slower than that of oF1PEA (**Supplementary Figure 7b**). This suggests that when n = 1 phase was excited, the excitons can transfer their energy to n = 2 phase in the case of PEA, mF1PEA and pF1PEA based 2D OIHP films. In contrast, oF1PEA sample shows the quickest decay of the absorption intensity (n = 2), indicating that such energy transfer (from n = 1 to n = 2) is not significant. Furthermore, for oF1PEA based 2D OIHP film, the signal for the 3D phase in the full spectrum (**Fig. 2e**) has a significant increase of intensity from 1 ps to 100 ps: we suspect that there is an efficient energy transfer from n=1 phase to the 3D phase due to their proximity, consistent with the PL spectrum for oF1PEA based 2D perovskite (**Fig. 2b**).

Supplementary Note 3

ToF-SIMS analyses were conducted using a ToF SIMS V (ION TOF, Inc. Chestnut Ridge, NY) instrument with a Cs^+ sputtering gun.

We tried elemental profiling method (ToF-SIMS), to further confirm the proposed phase distribution by analyzing F and organic fragments from spacer cations (**Supplementary Figure 8**). However, we could only observe a clear trend for mF1PEA 2D OIHP (**Supplementary Figure 8c**). For the rest, the substrate signal (Si) appeared at the very early stage, indicating a high etching rate (**Supplementary Figure 8 a, b, d**). Therefore, we could not draw a clear conclusion from ToF-SIMS result.

Supplementary Note 4

For UPS measurement, films of the OIHPs were deposited on ITO substrate by spin coating. The precursor solution contains 0.25 M PbI_2 and 0.5 M ammonium iodide salt (i.e., PEAI or F1PEAI) and 9:1 DMF/DMSO is used as solvent. The precursor solutions were spun at 5000 rpm for 20 s and followed by annealing at 80 °C for 5 minutes. 3D perovskite ($MAPbI_3$) was deposited following a previous reported procedure.[6] The kinetic energy was probed by Kratos Axis Ultra DLD Ultraviolet Photoelectron Spectrometer.

We conducted ultraviolet photoelectron spectrometer (UPS) on the film with pure n = 1 phases, i.e., PEA_2PbI_4, $oF1PEA_2PbI_4$, $mF1PEA_2PbI_4$, and $pF1PEA_2PbI_4$, and 3D phase ($MAPbI_3$) to study the energy levels of these perovskite phases. Because of the gradient structure transition from n = 1 to 3D, we believe that other 2D phase such as n = 2, 3, 4, etc. should have the energy levels lying between n = 1 and 3D. As shown in **Supplementary Figure S9** and **Supplementary Table 4**, the valence band of these n = 1 2D OIHP ranges from -5.8 eV to -6.4 eV. As comparison, we also conducted UPS on 3D perovskite samples and the valence band position is 5.8 V. Therefore, it is highly possible that a type I band alignment formed in our 2D OIHP films. However, it is worth noting that our 3D perovskite has a different valence band position compared to the work reported by others (~-5.4 eV) and UPS is a highly surface sensitive technique and the surface of perovskite films could suffer from contamination and degradation. Therefore, it’s hard to draw the conclusion the real band alignment in our 2D OIHP films with multiple phases.

Supplementary Note 5

The $T = 0$ K formation enthalpies were estimated using density functional theory (DFT) calculated total energies as implemented in VASP[7, 8] by way of $\Delta H_f^{0K} \approx E_{A2PbI4} - (2E_{AI} + E_{PbI2})$. DFT calculations used the Projector Augmented Wave (PAW) method[9, 10] to describe the effects of core electrons and Perdew−Burke− Ernzerhof (PBE)[11] implementation of the Generalized Approximation (GGA) for the exchange-correlation functional. The energy cutoff was set to 530 eV for the plane-wave basis of the valence electrons. The optB86b-vdW functional[12] for dispersion corrections was applied. The total energy tolerance for the electronic energy minimization was 10^{-4} eV; for structure optimization, forces were minimized such that all atoms experience forces < 0.05 eV $Å^{-1}$ after relaxation of the ionic coordinates and unit cell shape and volume. The product structures were idealized if the crystal structures were solved with split-site disorder, as discussed in the supporting information. The structures of fluorinated phenylethyl ammonium-iodide salts used for formation enthalpy calculations were obtained by relaxation of the reported structure of phenylethylammonium bromide,[13] after substitution of the bromine by iodine and relevant hydrogen with fluorine.

The formation enthalpies calculated for all the four 2D OIHPs are positive. Considering the relative small entropies change for solid state reaction, this suggests that 2D OIHP ($n = 1$) materials are not thermodynamically stable. However, this does not account for systematic errors associated with the exchange-correlation functional and finite temperature. For instance, the enthalpies might be temperature-dependent and entropic considerations may not be negligible. Last, highly idealized starting structures may also introduce systematic errors. Nevertheless, the trend of formation enthalpies is consistent with the quality of single crystal (pF1PEA2PbI4 ≈ mF1PEA2PbI4 > PEA2PbI4 >> oF1PEA2PbI4, Figure S4) and also consistent with the structure transition energy reported by Li et al.[14] Therefore, we report only the relative differences in formation energies, to approximate a fortuitous cancellation of systematic errors (**Fig. 6**).

Supplementary Discussion

Supplementary Discussion 1

We performed a thin film X-ray diffraction (XRD) experiment shown in **Supplementary Figure 11 and 12**. In the XRD profiles, we observed peaks of strong intensity around 14.2° and 28.5° in all samples, consistent with previous work on similar 2D OIHP films.[15, 16] Since our films contain multiple phases (both 2D and 3D phases) and these two peaks (14.2° and 28.5°) are present in XRD data for both 3D (**Supplementary Figure 12**) and 2D OIHPs, they can either be labelled as (111) and (202) based on 2D OIHP structure[15, 17] or (110) and (220) based on 3D OIHP structure.[18] Here we labelled them as (110) and (220) for our discussion. The absence of XRD peaks below 10° in PEA or pF1PEA 2D OIHP films indicates that these two films do not have many 2D OIHP crystalline phases with the inorganic layers parallel to the substrate.[19, 20] In contrast, a family of peaks from 2D phase(s) are strongly visible in the oF1PEA 2D OIHP film. Based on our single crystal test, these peaks belong to the (002) family of peaks from $n = 1$ phase. For the mF1PEA 2D OIHP film, we observe a stronger (002) family of peaks from 2D phases when compared to PEA and pF1PEA 2D OIHP films, but they are still weaker than those in oF1PEA 2D OIHP film. This indicates that there are 2D phases with the inorganic layers parallel to the substrate in mF1PEA 2D OIHP film, more than those in PEA and pF1PEA 2D OIHP film, but less than those in oF1PEA 2D OIHP film. We will discuss the crystal orientation further with the results from grazing incidence wide angle X-ray scattering (GIWAXS, *vide infra*) of all these 2D OIHP films. In addition to the different peak intensity for each film, the full-width at half-maximum (FWHM) for both the (110) and (220) peaks of all four samples are plotted in **Supplementary Figure 13**. In sharp contrast with other films, the FWHMs of oF1PEA 2D OIHP are larger than those of the rest. This suggests that the crystallinity of the oF1PEA 2D OIHP film is much worse than the other three films. This further explains the low photovoltaic performance of oF1PEA 2D OIHP.

Supplementary Discussion 2

SCXRD data for pF1PEA, mF1PEA, and oF1PEA were collected at room temperature using a Bruker D8 Quest ECO diffractometer equipped with a microfocus Mo K radiation source and Photon 50 CMOS half-plate detector. Single crystals were mounted onto a glass fiber with 5-minute epoxy. Bruker SAINT was used for integration and scaling of collected data and SADABS (multi-scan) was used for absorption correction. Starting models for the three compounds were generated using the intrinsic phasing method in SHELXT.[21] SHELXL2014 was used for least-squares refinement.[22] Structures, including disorder, based on electron densities are provided, as well as idealized supercells without disorder based on chemically-reasonable bond distances and molecular configurations.

$pF1PEA_2PbI_4$ has a fully ordered structure with no split atomic positions, indicating long range order for both the inorganic $[PbI_6]$ and organic pF1PEA units, and that registry between the inorganic layers is retained. The aromatic moieties within the organic interlayer gallery face the same direction resulting in a co-aligned configuration (**Fig. 5d** and **5h** of the main text). This leads to short intermolecular contacts between molecules within the top and bottom layers of the interlayer gallery of 2.731 Å (H5···F1) and 2.816 Å (F1···H7). Additionally, within the top and bottom layer, neighboring pF1PEA molecules have relatively short H5···H4 contacts of 2.872 Å.

The mF1PEA molecules within $mF1PEA_2PbI_4$, on the other hand, pack in a different manner than pF1PEA in $pF1PEA_2PbI_4$. The crystal structure results in significant disorder that is crystallographically accommodated using a split-occupancy of atoms to capture the total electron density. However, using the electron density of the heavy atoms, the relationship to other compounds, and realistic constraints on bond distances, we have assembled an idealized structural model without disorder that describes how the mF1PEA molecules are packed in the lattice. A significant difference between mF1PEA and pF1PEA is the presence of split axial iodine atomic positions and consequentially a splitting of the ammonium groups on the mF1PEA molecules. This disorder is similar to that observed in $(3\text{-FPEA})_2SnI_4$ where the splitting of sites was attributed to loss of registry between inorganic sheets within the material. This disorder results in a superimposed "average" structure of the two possible octahedral tilting configurations that can be adopted in ~50% probability (**Supplementary Figure 16a**). If one assumes that the lead is octahedrally coordinated in a relatively regular octahedron (as found in nearly all related compounds), then one finds the same tilting pattern found in $pF1PEA_2PbI_4$.

In addition to the split iodine positions, there are split positions of the mF1PEA molecules in the crystallographically-rigorous model. However, in the disordered structural model, for each of the iodine positions at a given split-site, there is a specific $R\text{-}CH_2\text{-}NH_3$ orientation that corresponds with realistic interatomic distances (**Supplementary Figure 16b**). This is due to the ammonium group always pointing towards the "puckered-out" orientation of the Pb-I-Pb bonds (cf., **Supplementary Figure 17**); the intermolecular contacts for an ammonium group facing towards the "puckered-in" direction are too short to be realistic. As neighboring octahedra must tilt-in and

then out to retain its structure (**Supplementary Figure 17**), the orientations of the R-CH_2-NH_3 cations must also alternate between the two possible configurations to ensure the bond distances between the inorganic and organic units are realistic. Therefore, we have generated an idealized supercell model in space group *P*1 that has the chemically-reasonable orientations included for both the [PbI_6] inorganic framework and the mF1PEA molecules. Within a single layer of the organic interlayer gallery, neighboring aromatic moieties are rotated relative to one another to generate a herringbone configuration (**Fig. 6c**). Compared to pF1PEA, this packing arrangement leads to even shorter intermolecular contacts within a layer (H2B···F1 of 2.512 Å), but leads to longer contacts between molecules in the top and bottom of the interlayer gallery of 3.005 Å (H6···F1).

Lastly, oF1PEA$_2$PbI$_4$ represents yet another type of packing and disorder that is different from pF1PEA$_2$PbI$_4$ and mF1PEA$_2$PbI$_4$. The crystal structure, as solved, is again an "average" structure with split sites such as those seen in mF1PEA$_2$PbI$_4$; however, there is no site splitting within the inorganic framework (i.e., each atom is fully occupied). Instead, the disorder arises from the different orientations the aromatic moieties can adopt, which are then coupled with the ammonium groups. There are two possible orientations the oF1PEA cations can adopt within a single layer, as illustrated in red and blue in **Supplementary Figure 17**. Interatomic distances dictate that the aromatic moieties must orient in a colinear fashion (**Fig. 5b**, similar to pF1 PEA$_2$PbI$_4$), but the neighboring layer along the stacking direction must then be oriented ~90° relative when projecting along the c-axis (**Fig. 6b** and **6f**). This is further illustrated in **Supplementary Figure 17** where either the red or blue molecules exist in a single layer, but both colors cannot exist at the same time. Because the aromatic moieties orient in this motif, and the fact that the R-CH_2-CH_2-NH_3 groups prefer the "J-shaped" gauche conformation relative to the inorganic sub-structural unit, the total number of possible orientations for the ammonium group is three, rather than four as seen in mF1PEA$_2$PbI$_4$. This is because the aromatic groups orient colinearly, and thus are always facing the same direction. This hinders the organic cations near the "puckered in" I-Pb-I geometry and allows us to assign the specific colors to the differently oriented molecules (**Supplementary Figure 17**). The occupancies of each molecular orientation were allowed to freely refine, which revealed a nearly perfect 50% occupancy for either of the two possible configurations (red or blue in **Supplementary Figure 17**). This indicates that like in mF1PEA$_2$PbI$_4$, each possible configuration occurs in the same total amount; however, the configurations lack ordering along the *c*-axis (**Supplementary Figure 18**). An idealized superstructure in P1 was also generated for oF1PEA$_2$PbI$_4$, which was used for DFT calculations.

Supplementary References

1. Kim, J.Y., *et al.* Efficient Tandem Polymer Solar Cells Fabricated by All-Solution Processing. *Science* **317**, 222-225 (2007).

2. Gilot, J., Wienk, M.M., Janssen, R.A.J. Measuring the External Quantum Efficiency of Two-Terminal Polymer Tandem Solar Cells. *Adv. Funct. Mater.* **20**, 3904-3911 (2010).

3. Burdick, J., Glatfelter, T. Spectral Response and I–V Measurements of Tandem Amorphous-Silicon Alloy Solar Cells. *Solar Cells* **18**, 301-314 (1986).

4. Dou, L., *et al.* Tandem Polymer Solar Cells Featuring a Spectrally Matched Low-Bandgap Polymer. *Nat. Photonics* **6**, 180 (2012).

5. Liu, F., Yan, C., Sun, K., Zhou, F., Hao, X., Green, M.A. Light-Bias-Dependent External Quantum Efficiency of Kesterite Cu_2ZnSnS_4 Solar Cells. *ACS Photonics* **4**, 1684-1690 (2017).

6. Deng, Y., Zheng, X., Bai, Y., Wang, Q., Zhao, J., Huang, J. Surfactant-Controlled Ink Drying Enables High-Speed Deposition of Perovskite Films for Efficient Photovoltaic Modules. *Nat. Energy* **3**, 560-566 (2018).

7. Kresse, G., Hafner, J. Norm-Conserving and Ultrasoft Pseudopotentials for First-Row and Transition Elements. *J. Phys. Condens. Matter* **6**, 8245 (1994).

8. Kresse, G., Furthmüller, J. Efficient Iterative Schemes for Ab Initio Total-Energy Calculations Using a Plane-Wave Basis Set. *Phys. Rev. B* **54**, 11169-11186 (1996).

9. Blöchl, P.E. Projector Augmented-Wave Method. *Phys. Rev. B* **50**, 17953-17979 (1994).

10. Kresse, G., Joubert, D. From Ultrasoft Pseudopotentials to the Projector Augmented-Wave Method. *Phys. Rev. B* **59**, 1758-1775 (1999).

11. Perdew, J.P., Burke, K., Ernzerhof, M. Generalized Gradient Approximation Made Simple. *Phys. Rev. Lett* **77**, 3865-3868 (1996).

12. Klimeš, J., Bowler, D.R., Michaelides, A. Van der Waals Density Functionals Applied to Solids. *Phys. Rev. B* **83**, 195131 (2011).

13. Rademeyer, M. 2-Phenylethylammonium Bromide. *Acta Crystallogr. E* **63**, o221-o223 (2007).

14. Li, T., Dunlap-Shohl, W.A., Han, Q., Mitzi, D.B. Melt Processing of Hybrid Organic–Inorganic Lead Iodide Layered Perovskites. *Chem. Mater.* **29**, 6200-6204 (2017).

15. Tsai, H., *et al.* High-Efficiency Two-Dimensional Ruddlesden–Popper Perovskite Solar Cells. *Nature* **536**, 312-314 (2016).

16. Smith, I.C., Hoke, E.T., Solis-Ibarra, D., McGehee, M.D., Karunadasa, H.I. A Layered Hybrid Perovskite Solar-Cell Absorber with Enhanced Moisture Stability. *Angew. Chem. Int. Ed.* **53**, 11232-11235 (2014).

17. Stoumpos, C.C., *et al.* Ruddlesden–Popper Hybrid Lead Iodide Perovskite 2D Homologous Semiconductors. *Chem. Mater.* **28**, 2852-2867 (2016).

18. Baikie, T., *et al.* Synthesis and Crystal Chemistry of the Hybrid Perovskite $(CH_3NH_3)PbI_3$ for Solid-State Sensitised Solar Cell Applications. *J. Mater. Chem. A* **1**, 5628-5641 (2013).

19. Xu, Z., *et al.* Phase Transition Control for High Performance Ruddlesden–Popper Perovskite Solar Cells. *Adv. Mater.* **30**, 1707166 (2018).

20. Venkatesan, N.R., Labram, J.G., Chabinyc, M.L. Charge-Carrier Dynamics and Crystalline Texture of Layered Ruddlesden–Popper Hybrid Lead Iodide Perovskite Thin Films. *ACS Energy Lett.* **3**, 380-386 (2018).

21. Sheldrick, G. SHELXT - Integrated Space-Group and Crystal-Structure Determination. *Acta Crystallogr. A* **71**, 3-8 (2015).

22. Sheldrick, G. Crystal Structure Refinement with SHELXL. *Acta Crystallogr. C* **71**, 3-8 (2015).
